# Targeting immunosuppressive Ly6C+ classical monocytes reverses anti-PD-1/CTLA-4 immunotherapy resistance

**DOI:** 10.3389/fimmu.2023.1161869

**Published:** 2023-06-28

**Authors:** B. Leticia Rodriguez, Limo Chen, Yanli Li, Shucheng Miao, David H. Peng, Jared J. Fradette, Lixia Diao, Jessica M. Konen, Frank R. Rojas Alvarez, Luisa M. Solis, Xiaohui Yi, Aparna Padhye, Laura A. Gibson, Joshua K. Ochieng, Xiaofei Zhou, Jing Wang, Don L. Gibbons

**Affiliations:** ^1^ Department of Thoracic/Head and Neck Medical Oncology, The University of Texas MD Anderson Cancer Center, Houston, TX, United States; ^2^ Department of Obstetrics and Gynecology, Shanghai General Hospital, Shanghai Jiao Tong University, School of Medicine, Shanghai, China; ^3^ United of Texas (UT) Health Graduate School of Biomedical Sciences, The University of Texas MD Anderson Cancer Center, Houston, Texas, United States; ^4^ Department Bioinformatics and Computational Biology, The University of Texas MD Anderson Cancer Center, Houston, TX, United States; ^5^ Department of Translational Molecular Pathology, The University of Texas MD Anderson Cancer Center, Houston, TX, United States; ^6^ Bellicum Pharmaceuticals, Inc., Houston, TX, United States; ^7^ Department of Immunology, The University of Texas MD Anderson Cancer Center, Houston, TX, United States; ^8^ Department of Molecular and Cellular Oncology, The University of Texas MD Anderson Cancer Center, Houston, TX, United States

**Keywords:** monocytes, immunotherapy, resistance, myeloid cells, PD-1/CTLA-4 immunotherapy

## Abstract

**Introduction:**

Despite significant clinical advancement with the use of immune checkpoint blockade (ICB) in non-small cell lung cancer (NSCLC) there are still a major subset of patients that develop adaptive/acquired resistance. Understanding resistance mechanisms to ICB is critical to developing new therapeutic strategies and improving patient survival. The dynamic nature of the tumor microenvironment and the mutational load driving tumor immunogenicity limit the efficacy to ICB. Recent studies indicate that myeloid cells are drivers of ICB resistance. In this study we sought to understand which immune cells were contributing to resistance and if we could modify them in a way to improve response to ICB therapy.

**Results:**

Our results show that combination anti-PD-1/CTLA-4 produces an initial antitumor effect with evidence of an activated immune response. Upon extended treatment with anti-PD-1/CTLA-4 acquired resistance developed with an increase of the immunosuppressive populations, including T-regulatory cells, neutrophils and monocytes. Addition of anti-Ly6C blocking antibody to anti-PD-1/CTLA-4 was capable of completely reversing treatment resistance and restoring CD8 T cell activity in multiple KP lung cancer models and in the autochthonous lung cancer Kras^LSL-G12D^/p53^fl/fl^ model. We found that there were higher classical Ly6C+ monocytes in anti-PD-1/CTLA-4 combination resistant tumors. B7 blockade illustrated the importance of dendritic cells for treatment efficacy of anti-Ly6C/PD-1/CTLA-4. We further determined that classical Ly6C+ monocytes in anti-PD-1/CTLA-4 resistant tumors are trafficked into the tumor via IFN-γ and the CCL2-CCR2 axis. Mechanistically we found that classical monocytes from ICB resistant tumors were unable to differentiate into antigen presenting cells and instead differentiated into immunosuppressive M2 macrophages or myeloid-derived suppressor cells (MDSC). Classical Ly6C+ monocytes from ICB resistant tumors had a decrease in both Flt3 and PU.1 expression that prevented differentiation into dendritic cells/macrophages.

**Conclusions:**

Therapeutically we found that addition of anti-Ly6C to the combination of anti-PD-1/CTLA-4 was capable of complete tumor eradication. Classical Ly6C+ monocytes differentiate into immunosuppressive cells, while blockade of classical monocytes drives dendritic cell differentiation/maturation to reinvigorate the anti-tumor T cell response. These findings support that immunotherapy resistance is associated with infiltrating monocytes and that controlling the differentiation process of monocytes can enhance the therapeutic potential of ICB.

## Graphical Abstract

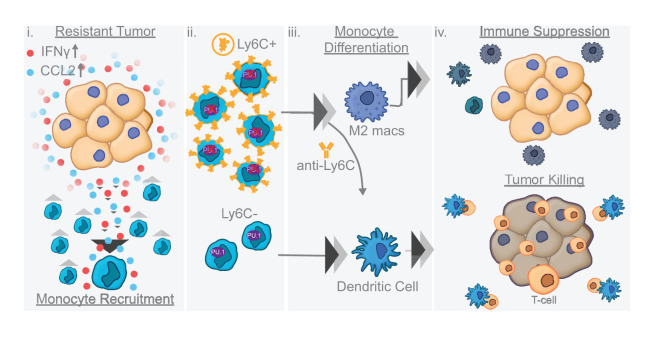

## Introduction

Immunotherapy has considerably changed the treatment landscape for patients with lung cancer over the past decade. Multiple checkpoint inhibitors have been FDA approved alone, in combination (anti-PD-(L)1/CTLA-4) or with chemotherapy for NSCLC in the locally-advanced or metastatic setting including nivolumab, pembrolizumab, durvalumab, atezolizumab, ipilimumab, and tremelimumab (1–7). Checkpoint inhibitors have recently been approved for early-stage disease and are expected to make remarkable improvements in NSCLC patient survival (8, 9). Efforts are now geared towards expanding the use of ICB to benefit the largest number of patients, because despite the remarkable clinical improvements, a major subset of patients do not respond to therapy due to adaptive or acquired resistance (10). The dynamic nature of the tumor microenvironment and the mutational load driving tumor immunogenicity limit the efficacy to ICB (11). Since the majority of NSCLC patients will eventually progress on ICB therapy, the exploration of novel concurrent or sequential combinatorial treatment approaches is essential.

There has been increasing evidence showing that myeloid cells (MC) are a potential driver of ICB resistance (12, 13). The MC population includes granulocytes, monocytes, macrophages and dendritic cells, which are far more abundant than T cells in tumors (14). They are considered critical modulators of the immune tumor microenvironment due to their dynamic differentiation capacity, complex specialized function and polarization (15). Here we describe a novel strategy of modulating the differentiation capacity of a progenitor myeloid cell population, namely monocytes, into dendritic cells and attaining durable therapeutic benefit to ICB resistance in lung cancer models.

We found that adaptive resistance to combination anti-PD-1/CTLA-4 was due in part to immunosuppressive cells including regulatory T cells, neutrophils, and monocytes. Addition of Ly6C blockade to the anti-PD-1/CTLA-4 combination was critical to prevent resistance from occurring and restored T cell activity. Importantly blockade of Ly6C is sufficient to change the differentiation capacity of the monocyte subsets into an anti-tumor dendritic cell population. The classical/nonclassical monocyte subsets identified have diverse differentiation potential. Our study identified that classical Ly6C+ monocytes in ICB resistant tumors have loss of Flt3/PU.1, which prevents their differentiation into dendritic cells. We show for the first time that blocking Ly6C on an intratumoral progenitor monocyte can enhance immunotherapy and ultimately influence the ability of the immune system to durably control cancer cells.

## Materials and methods

### Reagents and cancer cell lines

Anti-mouse PD-1 (clone RMP1-14), anti-mouse CTLA-4 (clone 9D9), anti-mouse CD80 (clone 16-10A1), anti-mouse CD86 (clone GL-1), anti-mouse Ly6C (clone Monts 1), anti-mouse CCL2 (clone 2H5), and the isotype-matched IgG controls were purchased from BioXCell. Recombinant murine CCL2 (catalog #250-10), IFN-γ (catalog #315-05), IL4 (catalog #214-14), GM-CSF (catalog #315-03), M-CSF (catalog #315-02) were purchased from PeproTech. Murine lung cancer cell lines 393P, 412P, 344P, 344SQ, and 307P were derived from *K-ras^LA1/+^p53^R172HΔg/+^* mice as previously described in multiple prior lung cancer studies (16–23). The LLC-JSP Lewis lung tumor cell line as well as the B16 and B16-OVA melanoma cell lines were maintained in our laboratories (24, 25).

### Lung cancer patient samples and mRNA expression profiling

Experimental details regarding TCGA datasets including RNA extraction, mRNA library preparation, sequencing (Illumina HiSeq platform), quality control, data processing and quantification of gene expression are previously published (26). For the PROSPECT and BATTLE-2 samples, the mRNA was extracted from frozen tumor tissue corresponding to the same specimen from which the formalin fixed, paraffin embedded blocks were made. Array based expression profiling of PROSPECT tumors was performed using the Illumina Human WG-6 v3 BeadChip, according to the manufacturer’s protocol. Gene expression data for the PROSPECT dataset have been previously deposited in the GEO repository (GSE42127) (27, 28). The raw data files of transcriptomes were analyzed using Bioconductor R packages. Scatter plots were generated through the cBioPortal (29, 30).

### Animal tumor models

Animal studies were reviewed and approved by the University of Texas MD Anderson Cancer Center Institutional Animal Care and Use Committee (IACUC). Six- to 8-week-old 129/Sv, C57BL/6, OT-I TCR transgenic mice were obtained from Charles River Laboratories and maintained in our laboratories. CD11c-DTR mice were from Jackson Laboratories.

For studying the tumor growth and animal survival in syngeneic immunocompetent models, cancer cells with indicated numbers in 100 µl of culture medium were injected subcutaneously into the mouse flank. Tumor growth was measured with digital calipers once a week, and tumor sizes were calculated using the formula: ½ (length × width × width) at indicated time points. At end points, mice were sacrificed to examine tumor weight, metastatic lung nodules as described previously (17). The genetically engineered mouse models (GEMM) (Kras^LSL-G12D^/p53^fl/fl^) were generated as previously described (19, 31). Mice were infected at 4 months of age with a 2.5x10^7^ titer pfu adenovirus-cre by intratracheal intubation. Treatment with antibodies were performed at 2-3 months post-infections when tumors were visualized by micro-CT imaging. All mice were immunocompetent and assessed for health daily by the department of veterinary medicine and surgery at The University of Texas MD Anderson Cancer Center.

### *In vivo* treatments

**Figure 1:**
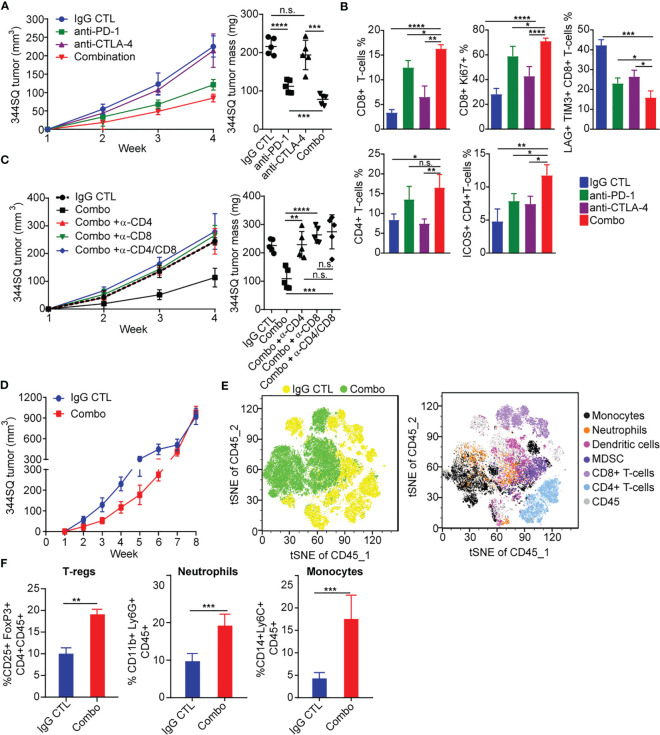
PD-1 and CTLA-4 blockade produces an initial antitumor effect with development of acquired resistance upon extended treatment **(A)** anti-PD-1 (200 µg per mouse), anti-CTLA-4 (200 µg per mouse), combination (200 µg of anti-PD-1 plus 200 µg of anti-CTLA-4), or their IgG control mixture was intraperitoneally injected into 129/Sv mice once a week for 3 weeks beginning on day 7 after 344SQ lung cancer cells were subcutaneously implanted (0.1 x 10^6^ cells per mouse). Tumors were measured once a week for 4 weeks. The tumor growth curves are shown in the left panel. Tumor weight at week 4 shown on right panel. **(B)** Tumors from **(A)** were harvested at the end point. The indicated immune markers were determined by FACS analysis and summarized data is shown as percentage of CD8, CD8+/Ki-67, Lag3+/Tim3+/CD8+, total CD4 T-cells, and ICOS+/CD4 T-cells. **(C)** 129/Sv mice were intraperitoneally injected with 400 µg per mouse of anti-CD4 (α -CD4), anti-CD8 (α -CD8), or anti-CD4 plus anti-CD8 (α -CD4/CD8) antibody when 344SQ cancer cells were injected. 200 µg of T cell depletion antibody per mouse was injected into the mice once weekly for 3 weeks to maintain T cell depletion. Mice were treated with combined anti-PD-1 and anti-CTLA-4 (Combination: 200 µg of anti-PD-1 plus 200 µg of anti-CTLA-4 per mouse) beginning on day 7 after a subcutaneous 344SQ cancer cell injection (0.1 x 10^6^ cells per mouse; n = 5) for 3 weeks. The tumor growth was monitored once a week for 4 weeks. The tumor growth curves are shown on the left and the tumor weights are shown on the right. **(D)** 129/Sv mice were treated weekly with combined anti-PD-1 and anti-CTLA-4 (Combination: 200 µg of anti-PD-1 plus 200 µg of anti-CTLA-4 per mouse) or their IgG control mixture (IgG control) beginning on day 7 after a subcutaneous 344SQ cancer cell injection (0.1 x 10^6^ cells per mouse; n = 5) for 7 weeks. The tumor growth was monitored once a week for 8 weeks. The tumor growth curves are shown. **(E)** tSNE CD45 plots shown from 344SQ tumors obtained from week 8 from IgG control or combo treated 129/Sv mice from **(D)**. **(F)** The enrichment of CD45^+^CD4^+^CD25^+^FoxP3^+^ T-Regulatory cells, CD45^+^CD3^-^CD11b^+^Ly6G^+^ neutrophils, and CD45^+^CD3^-^CD14+Ly6C^+^ monocyte cells in tumors were analyzed by FACS. *p* values were calculated with *t*-test. n.s., no significant difference; *p < 0.05; **p < 0.01; ***p < 0.001; ****p < 0.0001.

**Figure 2:**
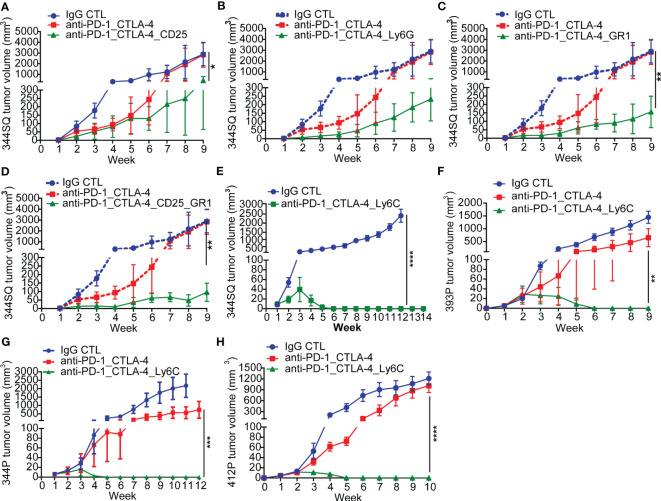
Anti-PD-1_CTLA-4 treatment resistance is eradicated by anti-Ly6C antibody treatment. **(A-H)** 129/Sv mice (n = 5) were treated weekly with indicated antibodies beginning on day 7 after a subcutaneous cancer cell injection (0.1 x 10^6^ 344SQ cells, 1 x 10^6^ 344P cells, 2 x 10^6^ 393P cells, or 1 x 10^6^ 412P cells per mouse). Dosage of all animal studies: 200 µg of anti-PD-1, 200 µg of anti-CTLA-4, 150 µg of anti-CD25, 300 µg of anti-Gr-1, 250 µg of anti-Ly6G, 200 µg of anti-Ly6C per mouse per injection. *p* values were calculated with *t*-test. n.s., no significant difference; *p < 0.05; **p < 0.01; ***p < 0.001; ****p < 0.0001.

**Figure 3:**
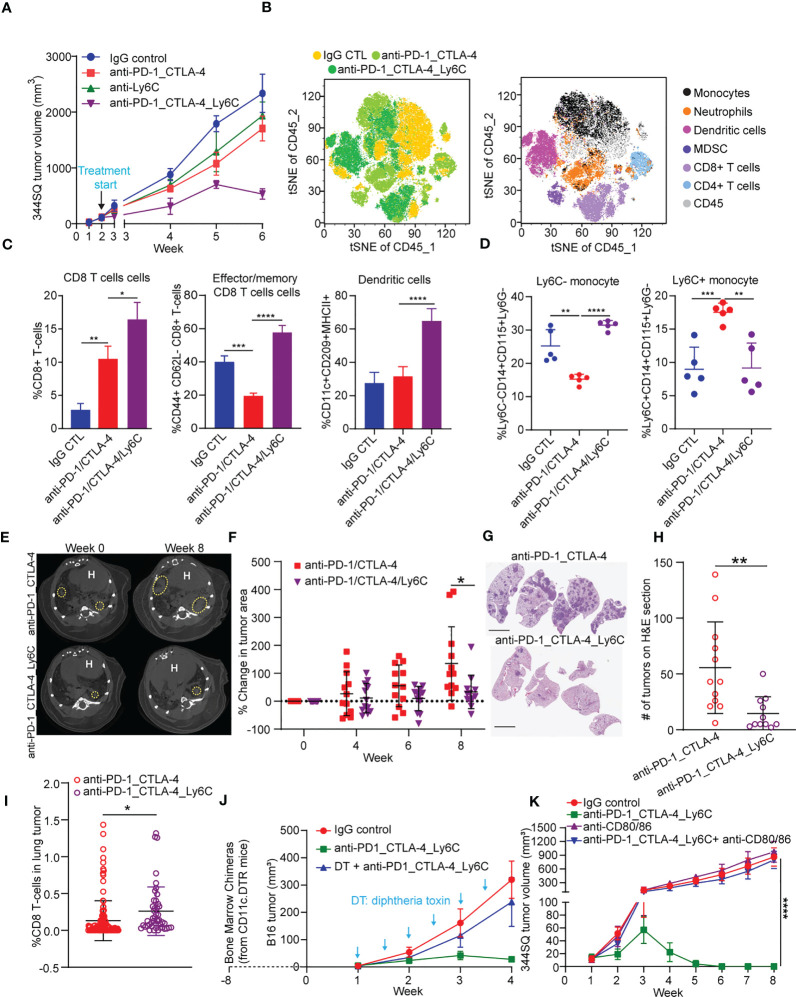
Anti-PD-1/CTLA-4/Ly6C treatment reduces tumor growth and increases CD8 T cells/dendritic cells. **(A)** 344SQ tumor bearing 129/Sv mice were weekly treated with anti-PD-1_CTLA-4 (200 µg of anti-PD-1 plus 200 µg of anti-CTLA-4 per mouse), anti-Ly6C alone (200 µg of anti-Ly6C per mouse), anti-PD-1_CTLA-4_Ly6C (200 µg of anti-PD-1 plus 200 µg of anti-CTLA-4 plus 200 µg of anti-Ly6C per mouse), or their IgG control mixture (IgG control) beginning on week 2 after a subcutaneous cancer cell injection (1 x 10^6^ cells per mouse; n = 5) for 4 weeks. Mice received total of 4 treatments starting at week 2 post tumor cell implantation. **(B)** tSNE CD45 plots from 344SQ tumors treated with IgG control, anti-PD-1_CTLA-4, or anti-PD-1_CTLA-4_Ly6C from **(A)**. Tumors from **(A)** were harvested to prepare single cell suspensions for FACS analysis. **(C)** Percentages of CD8 T cells (left), Effector/memory CD8 T cells (middle), and dendritic cells (right). **(D)** Percentage of Ly6C- (left) and Ly6C+ (right) CD14+CD115+ monocytes. Kras^LSL-G12D^/p53^fl/fl^ mice generated through intratracheal administration of adenovirus expressing Cre recombinase were treated with either anti-PD-1/CTLA-4 or anti-PD-1/CTLA-4/Ly6C for 8 weeks **(E)** Micro-CT images shown at week 0 (baseline) and week 8 (endpoint) for anti-PD-1/CTLA-4 or anti-PD-1/CTLA-4/Ly6C. Dashed yellow circles indicate lung tumors. H indicates of heart. **(F)** Percentage change of tumor area was calculated taking into account prior time point and normalized to the baseline measurement. **(G)** H&E stained lung sections at week 8 from anti-PD-1/CTLA-4 or anti-PD-1/CTLA-4/Ly6C treated mice from **(E)**. bar= 5 mm. **(H)** Number of lung tumors on H&E sections from **(G)** treated with anti-PD-1/CTLA-4 or anti-PD-1/CTLA-4/Ly6C weekly for 8 weeks. **(I)** IHC of CD8 stained lung tumors from **(F)** week 8. Percentage of CD8 T cells found in the lung tumors from Kras^LSL-G12D^/p53^fl/fl^ mice treated for 8 weeks with anti-PD-1/CTLA-4 or anti-PD-1/CTLA-4/Ly6C. **(J)** The dendritic cells from CD11c-DTR mice were transferred into C57BL/6 mice to generate the chimerical mice, after 8-week stabilization, the mice were treated with diphtheria toxin twice a week to maintain the depletion of dendritic cells. The B16 melanoma-bearing mice were treated weekly with anti-PD-1_CTLA-4_Ly6C for 3 weeks staring on week 1 after tumor cells inoculation. **(K)** 344SQ tumor bearing 129/Sv mice were treated weekly with blockade of PD-1, CTLA-4, and Ly6C (200 µg of anti-PD-1 plus 200 µg of anti-CTLA-4 plus 200 µg of anti-Ly6C per mouse) or their IgG control mixture (IgG control) beginning on day 7 after a subcutaneous cancer cell injection (0.1 x 10^6^ cells per mouse; n = 5) for 4 weeks. For blocking B7 signal, antibodies (anti-B7: 300 μg of anti-CD80 and 300 μg of anti-CD86 per mouse) were intraperitoneally administered 1 day before the first dose of therapy, and then once a week to maintain the blockade. ANOVA test was used to analyze the data. n.s., not significance; *p < 0.05; **p < 0.01; ***p< 0.001; ****p < 0.0001.

Mice were treated with antibodies (200 µg of anti-PD-1 per mouse; 200 µg of anti-CTLA-4 per mouse; 150 µg of anti-CD25 per mouse; 300 µg of anti-Gr-1 per mouse; 250 µg of anti-Ly6G per mouse; 200 µg of anti-Ly6C per mouse; 100 µg of anti-CCL2 per mouse; or combination) or their Ig G control via IP injection once a week for indicated weeks beginning on the indicated day after tumor cells were subcutaneously implanted. Mice were humanely euthanized at week 4 or week 8 (**Figure 1**). For the depletion experiments in **Figure 2** mice were treated until the IgG CTL group reached maximum allowable size per IACUC protocol. Mice were euthanized at week 9-14 depending on tumor growth from controls. For the anti-PD-1_CTLA4_Ly6C (**Figure 3A**) studies mice were euthanized at 6 weeks post implantation in order to evaluate immune cell infiltration. GEMM experiment (**Figures 3E–I**) were sacrificed at 8 weeks post infection when a few of the mice began to show signs of dyspnea/respiratory distress. B16 bone marrow chimeras experiment mice were scarified at week 4. The CD80/86 blockade experiment (**Figure 3K**) mice were euthanized at week 8.

### Chemotaxis assay

As modified as previously reported (32), the sorted Ly6C^high^ or Ly6C^low^ monocytes were performed using 24-well transwell plates (Cell Biolabs) with a 5 µm pore size polycarbonate filter. Monocytes were added to the upper chamber of the transwell (1 × 10^4^ cells/transwell) in the presence of CCL2 with indicated concentrations. Transwell plates were incubated at 37°C for 90 minutes. The transmigrated cells were collected from the lower chamber and counted on a flow cytometer. To determine the absolute number of cells in each sample, a standard number of 20 µM size fluorescent microspheres was added to each tube and counted along with the cells. The total number of transmigrated cells equals the number of counted monocytes × the total number of beads/the number of beads counted. The results are expressed as the mean ± SD of the chemotaxis index. The chemotaxis index represents the fold increase in the number of migrated cells in response to CCL2 over the spontaneous cell migration (to control medium).

### qRT-PCR assay

Total RNA was isolated from cultured cells or sorted cells from tumor tissues with TRIzol (Invitrogen). cDNA was synthesized by using the SuperScript III kit (Invitrogen), and qPCR was performed with the SYBR Green PCR Master Mix (Applied Biosystems). Primers were designed with National Center for Biotechnology Information (NCBI) primer design software. Relative expression levels were normalized by L32 and calculated by the 2^-ΔΔCt^ method (33). The primers used for amplification are found in the **Supplementary Table 1**.

### Enzyme-linked immunosorbent assay

To measure the concentration of IFN-α, IFN-γ, TNF-α, IL2, IL12 p40, IL23, IL27, and CCL2, tumor lysates or co-culture supernatants were used to perform ELISA assays with the kits for IFN-α (R&D Systems, 42120-1), IFN-γ (eBioscience, 88-7314-88), TNF-α (eBioscience, 88-7324-86), IL-2 (Thermo Fisher Scientific, BMS601), IL-12 p40 (abcam, ab171179), IL23 (R&D Systems, M2300), IL-27 (Thermo Fisher Scientific, BMS6024), and CCL2 (R&D Systems, DY479). The experiments were conducted in triplicates.

### Fluorescence-activated cell sorting analysis

Single-cell suspensions were prepared and stained according to standard protocols for flow cytometry including RBC lysis (Biolegend) following manufacturer recommendations. Cells were incubated with anti-CD16/32 to block nonspecific binding and then stained for 1 hr at room-temperature with appropriate dilutions of various combinations of fluorochrome conjugated antibodies. For intracellular staining, cells were fixed and permeabilized with BD Cytofix/Cytoperm (BD Biosciences). Cells were stained with intracellular mixture of fluorochrome conjugated antibodies for 30 minutes. The stained cells were acquired on a BD fortessa, Canto (BD Biosciences), or Cytek Aurora and the data was analyzed using FlowJo software (version 7.6; Tree Star). Doublets were excluded based upon forward and side scatter and dead cells were excluded by using BV510 Ghost dye. All flow cytometry plots shown and data quantitated from flow cytometry were gated upon doublet exclusion (FSC SSC) and viable (BV510 ghost -) CD45+ cells. Gating schemes found in (**Figure S10**). All antibodies used including dilutions are found in **Supplementary Table 1**.

### Monocyte differentiation assay

The assays were performed as previously described (32, 34–37). Briefly, isolated monocytes at 4 × 10^4^/mL were cultured in the 6-well plates with 10% fetal calf serum Iscoves modified Dulbecco medium containing designated cytokines. Cytokines were used at the following concentrations: 10 ng/mL of IL-4, 20 ng/mL of M-CSF, 100 ng/mL of GM-CSF, and 50 ng/mL of IFN-γ. After 6 days, cells were harvested to analyze the populations of myeloid-derived suppressor cells, M1 macrophages, and M2 macrophages by FACS. Dendritic cell maturation was induced by adding TNF-α (10 ng/mL) to the cultures on day 6. Mature dendritic cells were measured by FACS 2 days later.

### Bone marrow chimeras and diphtheria toxin treatment

The experimental protocol was modified according to a previous study (38). Briefly, femurs and tibias were taken from CD11c-DTR mice. The bone marrow was flushed out with a syringe and passed through a 70 μm nylon mesh to generate the single cell suspension. After red blood cells were lysed, the cells were washed twice and re-suspended (20 × 10^6^ cells/ml) in HBSS Supplementaryed with HEPES, L-glutamine, penicillin, streptomycin, and gentamycin sulfate. The recipient mice were irradiated (1000 rad) before 4 × 10^6^ bone marrow cells were intravenously transferred. The mice were allowed to rest for 8 weeks before B16 melanoma cell inoculation. Diphtheria toxin was intraperitoneally administered to mice at 4 ng/g body weight. Diphtheria toxin were administered 3-4 days apart to bone marrow chimeras for 6 times.

### Antibody-mediated T cell depletion and B7 blockade

For T cell depletion, mice were intraperitoneally pretreated with anti-CD4, anti-CD8, or combination antibodies (400 μg per mouse) one week before tumor cell injection. 200 μg of antibody per mouse was injected into the mice once weekly for indicated weeks beginning on day 1 after a subcutaneous cancer cell injection as previously described (18). For blockade of B7 signal, the protocol was modified according to the previous report (39). 300 μg of anti-CD80 and 300 μg of anti-CD86 per mouse were intraperitoneally administered 1 day before the first dose of combined anti-PD-1_CTLA-4, and then once a week to maintain the blockade.

### Statistical analysis

All statistical analyses were performed using GraphPad Prism version 6.00 (GraphPad Software, La Jolla). The un-paired t-test was used for comparisons between two-group means. The ANOVA test was used for comparisons between more than two-group means. All p values are two-tailed and for all analyses, p < 0.05 is considered statistically significant, unless otherwise specified.

## Results

### PD-1 and CTLA-4 blockade produce an initial antitumor effect with development of acquired resistance upon extended treatment

Due to the distinct signaling pathways of PD-1 and CTLA-4, we wanted to evaluate whether an antibody combination in NSCLC models would improve antitumor immunity as compared to single agent treatment. We evaluated a syngeneic KP tumor model (344SQ) derived from *Kras^LA1/+^; TP53^R172HΔG^* (KP) mice in the 129/Sv background (17, 19), a highly metastatic lung cancer model that has been previously shown to partially respond to PD-(L)1 blockade (17, 18, 40). We observed tumor growth inhibition with anti–PD-1 treatment, but not anti-CTLA-4, and enhanced tumor growth inhibition with combination blockade (**Figure 1A**). We further observed that both anti-PD-1 and combination blockade strongly increased T cell infiltration into and proliferation within the tumors, whereas CTLA-4 blockade alone did so to a lesser extent (**Figure 1B**). Combination PD-1/CTLA-4 blockade increased the proliferation and infiltration of CD4^+^ and CD8^+^ T-cells more than PD-1 blockade alone (**Figure 1B**), and significantly decreased the percentage of tumor infiltrating exhausted LAG3^+^ TIM3^+^ CD8^+^ T cells compared to either alone (**Figure 1B**). Single agent anti-PD-1 or anti-CTLA-4 increased the infiltrating ICOS^+^ CD4^+^ T cells to a similar degree, but combination blockade interestingly had a greater effect on the percentage of tumor-infiltrating lymphocytes (TILs) composed of ICOS^+^ CD4^+^T cells (**Figure 1B**). Evaluation of cytokine changes in the tumor microenvironment by ELISA-based analysis of tumor lysates demonstrated that combined blockade elevated the levels of cytokines IFN-γ, TNF-α, IL4, M-CSF, GM-CSF, and CCL2, while it suppressed the immune suppressive cytokine TGF-β (**Figure S1A**). The effect of anti-PD-1 on IFN-γ and TNF-α production was significantly stronger than that of anti-CTLA-4, while the effect on TGF-β, IL4, M-CSF, GM-CSF, and CCL2 was similar (**Figure S1A**).

To evaluate the contribution of either CD4^+^ or CD8^+^ T cells in the anti–PD-1/CTLA-4 combination, we tested the *in vivo* effects of CD4^+^ and/or CD8^+^ T cell depletion on syngeneic tumor growth. Response to combination anti-PD-1/CTLA-4 diminished when CD4^+^ T cells, CD8^+^ T cells, or both T cell subsets were depleted by antibody treatment (**Figure 1C**). Although dual depletion of CD4^+^ and CD8^+^ T cells trended toward a larger tumor size, there was no significant difference from the single CD4^+^ or CD8^+^ depletion (**Figure 1C**). Anti-PD-1/CTLA-4 treatment inhibits tumor progression in a CD4^+^ and CD8^+^ T cell-dependent manner, with CD4^+^ and CD8^+^ T cells working in an orchestrated fashion to control tumors.

Despite the enhanced response to anti–PD-1/CTLA-4 combination treatment, the effect was transient when observed over an extended treatment period (**Figure 1D**). Tumors in the treatment group ultimately developed adaptive resistance to the combination therapy and by ~7-8 weeks had primary tumor size similar to the controls. We analyzed harvested tumor tissue at the point of treatment resistance (week 8) using flow cytometry and found that the immunosuppressive populations of T-regulatory cells, neutrophils, and monocytes were highly enriched in the treatment resistant tumors (**Figures 1E, F**).

### Anti-PD-1/CTLA-4 treatment resistance is reversed by addition of anti-Ly6C antibody

In tumors with resistance to the anti-PD-1/CTLA-4 treatment, there was a significant enrichment of CD45^+^CD4^+^CD25^+^FoxP3^+^ T-Regulatory cells, CD45^+^CD3^-^CD11b^+^Ly6G^+^ neutrophils, and CD45^+^CD3^-^CD14^+^Ly6C^+^ monocytes (**Figure 1F**). These populations have been well documented as immune suppressive in cancer immunotherapy (41–43). We evaluated their role in the resistance to combination PD-1/CTLA-4 blockade by adding: anti-CD25 (clone PC61 for depleting CD25^+^ T-Regulatory cells (44)), anti-Gr1 (clone RB6-8C5 for depleting CD45^+^CD3^-^CD11b^+^Gr1^+^ myeloid cells (45)), or anti-Ly6G (clone 1A8 for depleting CD45^+^CD3^-^CD11b^+^Ly6G^+^ neutrophils (46)). In each case the resistance to anti-PD-1/CTLA-4 treatment was substantially inhibited (**Figures 2A-D**), demonstrating a role for multiple immune cell populations in the antitumor effect, but in each case the tumors demonstrated resistance and the ability to grow out over time.

Given that Ly6C was highly up-regulated on infiltrating monocytes in tumors with resistance to the anti-PD-1/CTLA-4 treatment, we tested whether Ly6C blockade could reverse the resistance. Strikingly, when we used the anti-Ly6C antibody (clone Monts 1) to block Ly6C, the resistance to anti-PD-1/CTLA-4 was completely suppressed in the aggressive KP 344SQ tumor model (**Figures 2E**, **S2A**). We tested the concurrent anti-PD-1/CTLA-4/Ly6C therapy in multiple other KP NSCLC animal models and consistently observed that the resistance to anti-PD-1/CTLA-4 treatment was reversed by anti-Ly6C antibody treatment in 393P, 344P, and 412P KP syngeneic models (**Figures 2F-H**, **S2A-D**).

To further understand the efficacy of this combination, we tested whether dual blockade of Ly6C/PD-1 or Ly6C/CTLA-4 could produce the same complete tumor regression as triple blockade. Although anti-Ly6C/CTLA-4 treatment achieved a significantly better outcome in terms of tumor growth and immune infiltration than that of anti-Ly6C/PD-1, dual blockade of Ly6C/CTLA-4 did not produce a curative effect (**Figures S3A, B**). Importantly, the anti-Ly6C antibody Monts 1 in combination with clodronate liposomes (a commonly used macrophage depleting agent), was reportedly used for *in vivo* macrophage depletion (47) (https://bxcell.com/product/ly6c/). However, although we observed that Monts 1 clearly binds to Ly6C (**Figure S4A**), the antibody treatment alone did not demonstrate depleting effects (**Figure S4B**).

Taken together, multiple factors contribute to the immunosuppressive tumor microenvironment and resistance to anti-PD-1/CTLA-4 treatment. However, CD14^+^ Ly6C^+^ monocytes play a dominant role in the acquired resistance to combination ICB therapy.

### Tumor-infiltrating monocyte-derived dendritic cells play a key role in maximizing the antitumor immunity of anti-PD-1/CTLA-4 treatment

Triple blockade of Ly6C, PD-1, and CTLA-4 resulted in complete tumor responses, thus precluding further analysis of tumor tissues to explore the underlying mechanism of response (**Figures 2E-H**). To address this, we increased the number of implanted tumor cells and started treatment at two weeks post implantation (**Figure 3A**). The triple blockade of Ly6C, PD-1, and CTLA-4 caused a significant tumor regression by week 6 (**Figure 3A**).

Multi-parameter spectral flow cytometry from 344SQ tumors treated with anti-PD-1/CTLA-4 or anti-PD-1/CTLA-4/Ly6C and harvested at week 6 revealed drastic changes to immune cell populations. We observed an increase in classical monocytes (CD45+CD3-CD14+Ly6G-Ly6C+) and neutrophils (CD45+CD3-CD11b+Ly6C-Ly6G+) in anti-PD-1/CTLA-4 treated tumors (**Figure 3B**). Anti-PD-1/CTLA-4/Ly6C tumors had a significant reduction of monocytes, neutrophils, and an increase in dendritic cells (CD45+CD11b-CD11c+MHCII+F4/80-CD209a+), as compared to anti-PD-1/CTLA-4 treated tumors. Other immune cell populations such as M1/M2 macrophages and MDSC showed no significant difference between treatment groups.

We found that anti-PD-1/CTLA-4/Ly6C treated tumors had increased percentages of CD8 T cells and effector/memory CD8 T cells (**Figure 3C**) as compared to anti-PD-1/CTLA-4. We found no difference in M1 or M2 macrophages as compared to anti-PD-1/CTLA-4 or IgG CTL (**Figure S5A**), but interestingly we observed increased dendritic cells in the anti-PD-1/CTLA-4/Ly6C treated tumors (**Figure 3C**). Intratumoral monocytes from anti-PD-1/CTLA-4/Ly6C treated tumors were composed of nonclassical ~30% Ly6C- and ~10% classical Ly6C+ monocytes, but with the anti-PD-1/CTLA-4 combination we observe a decrease in nonclassical monocytes Ly6C- monocytes and increase in classical Ly6C+ monocytes (**Figure 3D**). Intriguingly, addition of anti-Ly6C to the combination was capable of restoring the levels of classical and non-classical monocytes to that of the IgG treated controls.

We further tested the efficacy of the triple blockade of Ly6C/PD-1/CTLA-4 in a clinically relevant autochthonous lung cancer GEMM (Kras^LSL-G12D^/p53^fl/fl^) where tumors are induced through intratracheal administration of adenoviral-Cre recombinase (23, 48). Primary lung tumors developed ~8 weeks after infection and were confirmed by micro-CT imaging (**Figure 3E**, left panel). Once tumors were confirmed, mice were randomized and treated with anti-PD-1/CTLA-4 or anti-PD-1/CTLA-4/Ly6C for 8 weeks (**Figure 3E**, right panel). Micro-CT image analysis revealed that mice treated with anti-PD-1/CTLA-4/Ly6C had a smaller percentage change of lung adenocarcinomas compared to mice treated with anti-PD-1/CTLA-4 by week 8 (**Figure 3F**). H&E stained lung sections at week 8 revealed fewer lung tumors in the mice treated with anti-PD1-1/CTLA-4/Ly6C as compared to anti-PD-1/CTLA-4 (**Figures 3G, H**, **S5B**). To assess immune response in the autochthonous lung tumors from Kras^LSL-G12D^/p53^fl/fl^ mice treated with anti-PD-1/CTLA-4 or anti-PD-1/CTLA-4/Ly6C we probed for CD8 and quantified the percentage of CD8 cells within each tumor. We found a higher percentage of CD8 T cells in lung tumors from Kras^LSL-G12D^/p53^fl/fl^ mice treated with anti-PD-1/CTLA-4/Ly6C compared to anti-PD-1/CTLA-4 (**Figures 3I**, **S5C**). Overall, these data demonstrate the efficacy of combining anti-PD-1/CTLA-4/Ly6C immunotherapy to control KP lung tumor growth.

Lastly, we sought to test the importance of dendritic cells within anti-PD-1/CTLA-4/Ly6C treated tumors. Using the B16 melanoma tumor model we observed similar response/resistance to combination anti-PD-1/CTLA-4 treatment in the KP lung model, as well as infiltration of Ly6C^+^ monocytes (**Figure S6A**). After inoculation of mice with B16-OVA tumor cells, we injected OVA-pulsed *ex vivo* generated tumor monocyte-derived dendritic cells (TMDC) into the tumor-bearing mice at weeks 1 and 2. Compared with the anti-PD-1/CTLA-4 treatment alone, the anti-PD-1/CTLA-4 treatment plus TMDCs was significantly more potent in suppressing the tumor growth and inducing cytokines from tumor-infiltrating effector T cells and dendritic cells (**Figures S6B, C**). Second, to confirm the role of dendritic cells in the anti-PD-1/CTLA-4/Ly6C antitumor response, we performed an *in vivo* dendritic cell depletion assay. The dendritic cells from CD11c-DTR mice were transferred into C57BL/6 mice to generate chimeric mice. After 8-weeks to allow for reconstitution, mice were implanted with B16 tumor cells and treated with combination anti-PD-1/CTLA-4/Ly6C with or without addition of diphtheria toxin (DT) twice a week to deplete the dendritic cells. The results demonstrated that the B16 tumors from mice treated with diphtheria toxin lacked dendritic cells and the efficacy of the anti-PD-1/CTLA-4/Ly6C was lost (**Figure 3J**).

Dendritic cell-mediated B7 signaling plays a critical role in the antitumor immune response (39), and we further tested the role of antigen expressing cells such as dendritic cells and macrophages in the tumor regression with anti-PD-1/CTLA-4/Ly6C treatment. When B7 signaling (CD80/CD86) was blocked, the treatment effect on KP tumors was reversed and demonstrated no efficacy (**Figures 3K**, **S6D**). B7 signaling (CD80/CD86) are expressed on dendritic cells and macrophages. APC-mediated B7 signaling is critical for anti-PD-1/CTLA-4/Ly6C treatment mediated tumor regression. Taken together the data reveal that addition of Ly6C blockade to anti-PD-1/CTLA-4 was capable of suppressing Ly6C+ monocyte levels, restoring T cell responses and delineated a critical role for dendritic cells within the anti-PD-1/CTLA-4/Ly6C treated tumors.

### Classical Ly6C+ monocytes in anti-PD-1/CTLA-4 resistant tumors are mediated by IFN-γ and the CCL2-CCR2 axis

**Figure 4:**
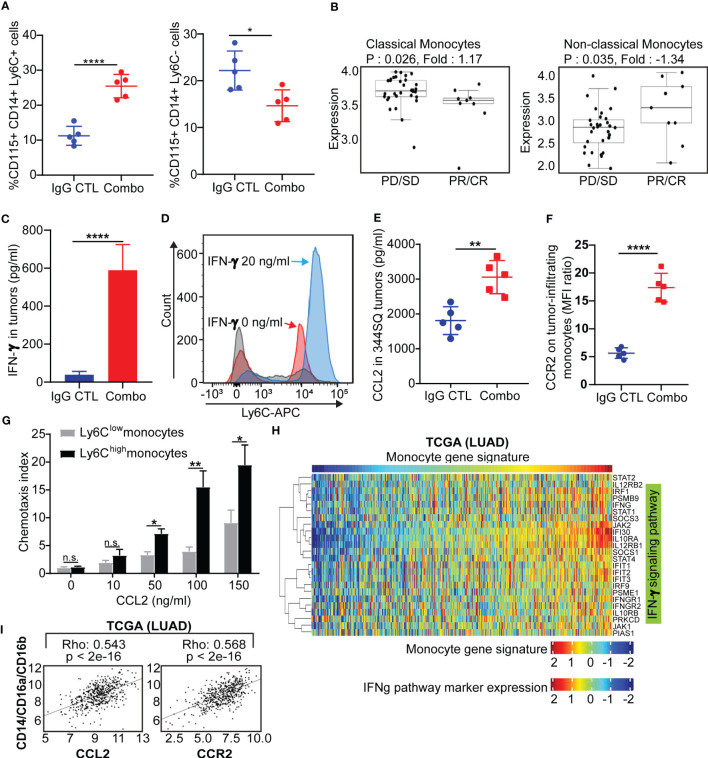
Classical Ly6C+ monocytes in anti-PD-1/CTLA-4 resistant tumors is mediated by IFN-γ and the CCL2-CCR2 axis **(A)** 129/Sv mice were treated weekly with combined anti-PD-1 and anti-CTLA-4 (Combination: 200 µg of anti-PD-1 plus 200 µg of anti-CTLA-4 per mouse) or their IgG control mixture (IgG control) beginning on day 7 after a subcutaneous 344SQ cancer cell injection (0.1 x 10^6^ cells per mouse; n = 5) for 7 weeks. FACS analysis from intertumoral monocytes CD115+CD14+ monocytes and Ly6C+ (left) or Ly6C- (right). **(B)** Classical monocytes and non-classical monocytes from melanoma nivolumab treated patients (GSE91061). **(C)** 129/Sv mice were treated weekly with combined anti-PD-1 and anti-CTLA-4 (Combination: 200 µg of anti-PD-1 plus 200 µg of anti-CTLA-4 per mouse) or their IgG control mixture (IgG control) beginning on day 7 after a subcutaneous 344SQ cancer cell injection (0.1 x 10^6^ cells per mouse; n = 5) for 7 weeks. The IFN-γ concentrations in tumors were measured by ELISA assay. **(D)** The monocytes were sorted (CD45^+^CD11b^+^CD115^+^Ly6G^-^ for sorting) from untreated 344SQ tumors. 10,000 monocytes were plated in the 12-well plate. After 12 hrs of IFN-γ (20 ng/ml) stimulation, the expression of Ly6C on monocytes was analyzed by FACS. The experiments were repeated at least three times. The representative histograms are shown. **(E)** The CCL2 concentrations in tumors treated with IgG CTL or combo treatment were measured by ELISA assay. **(F)** Monocytes from IgG CTL or combo treated 344SQ tumors were harvested at week 7 and FACS for analyzing CCR2 expression on total monocytes. **(G)** The sorted Ly6C^high^ or Ly6C^low^ monocytes from 344SQ tumors were added to the upper chamber of the transwell (1 × 10^4^ cells/transwell) in the presence of CCL2 with indicated concentrations. Transwell plates were incubated at 37 °C for 90 minutes. The transmigrated cells were collected from the lower chamber and analyzed by a flow cytometer. The chemotaxis index represents the fold increase in the number of migrated cells in response to CCL2 over the spontaneous cell migration. **(H)** Heat map of association between the mRNA levels of human monocyte gene signature and IFNγ pathway in the TCGA lung adenocarcinoma (n = 1008). Spearman correlation with adjusted p value < 0.05 was used as the criteria to select the most significant IFN-γ-related markers for generating the heat map. **(I)** Spearman’s rank correlation (Rho) was used to assess the association between CCL2/CCR2 and human monocyte marker expression in lung cancer patients’ samples from TCGA (LUAD). The results are shown with *t*-test. n.s., no significant difference; *p < 0.05; **p < 0.01; ****p < 0.0001.

Our data reveal that anti-Ly6C addition to the combination treatment can restore immune cell responses. How the monocytes are trafficked and which type of monocytes (classical vs. non-classical) is unknown. First, we evaluated Ly6C expression on CD115+ CD14+ monocytes within the IgG control or anti-PD-1/CTLA-4 treated tumors and found increased Ly6C+ expression and a decrease in Ly6C- on monocytes within combo treated tumors (**Figure 4A**). Monocytes are heterogenous and consist of a classical/intermediate Ly6C+CD11b+CD115+ population or a nonclassical Ly6C-CD11b+CD115+ population. Published single cell transcriptomic analysis of tumor-infiltrating myeloid cells in human and mouse lung cancers revealed that monocytes are conserved across the species with evidence of a one-to-one equivalence (15), with the murine Ly6C+ classical monocyte being equivalent to the human CD14++ classical monocyte. We used the human gene signatures from Zilionis et al. that are equivalent to the Ly6C+ classical and the Ly6C- non-classical monocytes and interrogated a dataset from melanoma patients treated with nivolumab (49). We found that classical monocytes were higher in patients showing progressive disease/stable disease as compared to patients showing partial response/complete response to immunotherapy and the reverse trend was found with the non-classical monocytes (**Figure 4B**). A similar trend was found in NSCLC patients treated with nivolumab or pembrolizumab, although not statistically different due to low sample numbers in the responder group (50) (**Figure S7A**). Overall, these data demonstrate that tumor-infiltrating Ly6C+ classical monocytes are associated with immunotherapy resistance.

It was previously reported that Ly6C is regulated by IFN-α, IFN-γ and IL-27 (51–53). We observed an increase of IFN-γ in tumors with both short-term and long-term anti-PD-1/CTLA-4 treatment but did not observe a significant change in IFN-α or IL-27 (**Figures 4C**, **S1A**). We therefore sought to determine whether anti-PD-1/CTLA-4-induced IFN-γ could regulate Ly6C on monocytes. Monocytes from the KP 344SQ tumors were sorted (CD45+CD11b+CD115+Ly6G-) and incubated with IFN-γ overnight. Ly6C expression on monocytes showed a significant up-regulation after IFN-γ stimulation (**Figure 4D**). Furthermore, CCL2 in tumors and CCR2 on tumor-infiltrating monocytes were increased with combination anti-PD-1/CTLA-4 treatment (**Figures 4E, F**, **S1A**). The CCL2-CCR2 axis plays an important role in recruiting monocytes into tumors (54) and we set up a chemotaxis assay to evaluate the CCL2-CCR2 axis in our models. CCL2-containing media was placed in the bottom chamber and sorted Ly6C^low^ or Ly6C^high^ monocytes were placed in the upper chamber. More Ly6C^high^ monocytes migrated in a manner that was CCL2 concentration dependent than Ly6C^low^ monocytes (**Figure 4G**). For *in vivo* testing we used an anti-CCL2 antibody, as previously reported (54, 55). The CCL2 blocking antibody treatment partially reversed the efficacy of the anti-PD-1/CTLA-4 treatment (**Figure S7B**), while significantly reducing intratumoral monocytes and mature dendritic cells (**Figures S7C, D**). Further analysis demonstrated that anti-CCL2 treatment dampened the T cell immune response to anti-PD-1/CTLA-4 therapy, with a decrease in total CD4 and CD8 T cell numbers and inhibition of T-cell proliferation (**Figure S7E**). Our data shows that IFN-γ stimulation leads to upregulation of Ly6C+ expression on CD115+ monocytes and that the Ly6C+ monocytes are recruited via the CCL2-CCR2 axis.

To investigate whether these observations occur in human lung cancers, we analyzed three independent lung cancer patient tumor datasets; The Cancer Genome Atlas (TCGA), PROSPECT and BATTLE-2 trials from MD Anderson (40). We utilized a monocyte gene signature as previously reported (56) and consistently found a strong correlation between the monocyte gene signature and the IFN-γ signature (57–59), (*p* < 0.05) (**Figures 4H**, **S8A**). A positive correlation was also found between monocytes (CD14^+^/CD16a^+^/CD16b^+^) and the CCL2-CCR2 axis (**Figures 4I**, **S8B**). Taken together, both murine and human data suggest that intertumoral monocyte infiltration is caused by signaling through the CCL2-CCR2 axis, which is potentiated by monocyte Ly6C levels regulated in an IFN-γ dependent manner.

### Anti-PD-1/CTLA-4 resistant monocytes have decreased differentiation capacity due to loss of Flt3/PU.1

**Figure 5:**
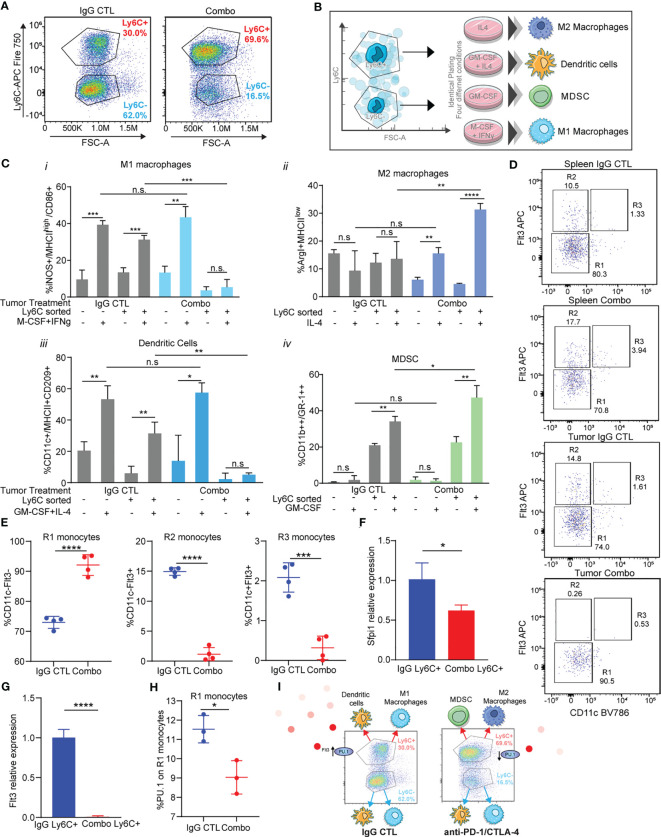
Immunotherapy resistant Ly6C+ monocytes have decreased antigen presenting cell differentiation capacity due to loss of Flt3/PU.1. **(A)** Tumor-infiltrating monocytes sorted from 344SQ tumors treated with IgG control or combo (anti-PD-1/CTLA-4) are classified into two populations: Ly6C^+^monocytes and Ly6C^-^monocytes. **(B)** The trans-differentiation assay scheme. **(C)** The differentiation assays were performed under different conditions. The percent of M1 macrophage, M2 macrophage, MDSC, or dendritic cell in total monocytes were analyzed with their markers by FACS at the end points. **(D)** heterogeneity of intertumoral Ly6C+ monocytes from 344SQ tumors treated with IgG CTL or combo (anti-PD-1/CTLA-4) for 7 weeks. Flt3 and CD11c flow dot plots were used to characterize Ly6C+ monocytes. R1, R2, and R3 gates are found in box gates. **(E)** Percentage of R1 monocytes CD11c-Flt3-, R2 monocytes CD11c-Flt3+, and R3 monocytes CD11c+Flt3+ from Ly6C+ intertumoral monocytes treated with IgG CTL or combo until week 7. **(F)** qPCR of Sfpi1 from Ly6C+ FACS sorted monocytes from IgG CTL or combo (anti-PD-1/CTLA-4) treated tumors. **(G)** qPCR of Flt3 from Ly6C+ FACS sorted monocytes from IgG CTL or combo (anti-PD-1/CTLA-4) treated tumors. **(H)** Intracellular percentage of PU.1 levels on R1 monocytes from Ly6C+ FACS sorted monocytes from IgG CTL or combo (anti-PD-1/CTLA-4) treated tumors. **(I)** schematic showing differentiation results from **(C)** and loss of Flt3/PU.1 in the combo (anti-PD-1/CTLA-4) resistant Ly6C+ monocytes. ANOVA test is shown. n.s., no significant difference; *p < 0.05; **p < 0.01; ***p < 0.001; ****p < 0.0001.

Monocytes are heterogeneous and function to differentiate into dendritic cells, macrophages, or MDSC based upon the cytokine milieu (54, 60–62). In the present study, we found that the tumor-infiltrating monocytes (CD45^+^CD11b^+^CD115^+^Ly6G^-^) could be classified into two subpopulations, Ly6C^+^ and Ly6C^-^ monocytes, with combination immunotherapy treated tumors showing higher percentage of Ly6C+ (**Figure 5A**). Our aim was to determine if Ly6C plays a role to determine monocyte differentiation or if Ly6C simply serves as a marker. To do so, we set up *ex vivo* monocyte differentiation assays according to previous reports (32, 34–37). Monocytes from KP tumors treated with IgG or anti-PD-1/CTLA-4 antibodies until week 7 were sorted using the CD45^+^CD11b^+^CD115^+^Ly6G^-^ markers and then further sorted into Ly6C^+^ and Ly6C^-^ subsets. The sorted monocytes with different expression levels of Ly6C were cultured *ex vivo* in 12-well plates containing various cytokine treatments (**Figure 5B**). M-CSF+IFNγ was added to the sorted monocytes to induce differentiation into M1 macrophages. IL-4 was added to induce differentiation into M2 macrophages. Dendritic cells were incubated with GM-CSF+IL-4 for 6 days then TNF-α was added for maturation. GM-CSF was added to induce differentiation into MDSC. Differentiated cells were then quantified using flow cytometry.

We observed that there was a higher percentage of M1 macrophage differentiation upon addition of M-CSF + IFNγ from monocytes sorted from IgG CTL treated tumors, as compared to monocytes treated with PBS (**Figure 5C**i). M1 macrophages differentiated from Ly6C- monocytes but not from the Ly6C+ monocytes sorted from combination treated tumors (**Figure 5C**i). M2 macrophage differentiation was not different across monocytes from IgG CTL tumors (**Figure 5C**ii). However, we observed changes in differentiation within the monocytes sorted from the combo treated tumors in which IL-4 had been added, with a higher percentage of M2 macrophages from the Ly6C+ vs the Ly6C- monocytes from the combo treated tumors (**Figure 5C**ii). Conversely, mature dendritic cells were obtained upon addition of cytokines to both Ly6C+ and Ly6C- monocytes from the IgG CTL treated tumor, but we did not observe dendritic cell differentiation from the Ly6C+ monocyte population from combo treated tumors, even though the Ly6C- monocyte population produced dendritic cell levels equivalent to the IgG CTL (**Figure 5C**iii). Finally, MDSC differentiation was performed with addition of GM-CSF. We found low levels of differentiation in the Ly6C- monocyte population from either IgG CTL or combo treated tumors (**Figure 5C**iv). Ly6C+ monocytes were the predominant contributor of MDSCs and the Ly6C+ monocytes from combo treated tumors had higher levels than the IgG CTL treated tumors (**Figure 5C**iv). These data suggest that Ly6C+ and Ly6C- monocytes from IgG CTL treated tumors differentiate into M1 macrophages and dendritic cells (**Figure 5C**). Ly6C- monocytes from combo treated tumors also differentiate into M1 macrophage and dendritic cells (**Figure 5C**), but the predominate Ly6C+ monocyte population found in combo treated tumors was unable to differentiate into M1 macrophages or dendritic cells and instead differentiated into M2 macrophages and MDSC (**Figure 5C**). The differences in differentiation potential are largely due to the Ly6C+ classical monocyte population found in the combination immunotherapy treated tumors.

We evaluated Ly6C+ monocyte heterogeneity to understand how polarization of classical Ly6C+ monocytes into macrophages or dendritic cells may be regulated (63). SIRPα+/Flt3+ staining on Ly6C+ monocytes can be subclassified into R1, R2, and R3 subpopulations that can differentiate into macrophages, dendritic cells, and pre-dendritic cells, respectively (63). We utilized a staining/gating scheme that included Flt3 and SIRPα to establish that the 344SQ tumor infiltrating Ly6C+ monocytes are heterogenous (**Figures 5D**, **S9A**). We found that IgG treated tumor-derived Ly6C+ monocytes had similar levels of R1, R2, and R3 monocytes as compared to matched spleens (**Figure 5D**). By contrast, the percentage of R1 tumor-derived monocytes was higher in combo treated as compared to IgG treated controls or matched spleens (**Figures 5D, E**), while the infiltrating monocytes from anti-PD-1/CTLA-4 treated tumors had decreased levels of R2 and R3 monocytes (**Figures 5D, E**). R2 and R3 monocytes give rise to monocyte-derived dendritic cells, whereas R1 monocytes generate macrophages. This data is consistent with the differentiation assay results (**Figure 5C**) demonstrating that Ly6C+ monocytes from the combo group have decreased levels of dendritic cells as compared to Ly6C+ monocytes from the IgG CTL group.

We next sought to understand whether the transcription factor *Sfpi1* (PU.1) was regulating the formation of R2 monocytes within the Ly6C+ subset. PU.1 is known to promote *Flt3* expression (64). We evaluated levels of *Spfi1* and *Flt3* in Ly6C+ sorted monocytes and found lower expression of both in the monocytes from combination treated tumors as compared to the IgG CTL (**Figures 5F, G**). There was also a decrease in the percentage of intracellular PU.1 in Ly6C+ monocytes from the combination treated resistant tumors (**Figure 5H**). It is known that Ly6C+ monocytes give rise to Ly6C- monocytes via Nr4a1 (Nur77) (65), with increased Ly6C+ monocytes in Nr4a1-/- mice. To assess for a role of Nr4a1 in tumor infiltrating monocytes we performed flow cytometry using intracellular Nur77 and qPCR from Ly6C sorted monocytes from combo resistant and IgG CTL treated tumors. We observed higher levels of Nur77 expression on the Ly6C- monocytes as compared to Ly6C+ (**Figure S9B**), while the Ly6C+ monocytes from combo treated tumors had similar Nur77 levels compared to the IgG CTL. Similarly, levels of Nr4a1 were elevated in the Ly6C- sorted monocytes from treated tumors and we found higher levels of Nr4a1 on combo treated Ly6C+ monocytes compared to IgG CTL tumors (**Figure S9B**). These data suggest that the increased infiltration of Ly6C+ monocytes in combo treated tumors was not due to a decrease in Nr4a1 (Nur77) expression. Taken together, these data suggest that in the combination therapy resistant tumors classical Ly6C+ monocytes have decreased Flt3/PU.1 expression (**Figure 5I**), which leads to their decreased ability to differentiate into antigen presenting cells and instead differentiate into immunosuppressive cells.

The data from multiple tumor models under treatment conditions that produced complete tumor eradication highlight a model in which the importance of tumor infiltrating monocyte-derived dendritic cells is critical to maximizing the therapeutic outcome of combined PD-1 and CTLA-4 blockade in lung cancer. Ly6C^+^ monocytes differentiate into immunosuppressive cell types, while their blockade drives dendritic cell differentiation/maturation to boost an anti-tumor T cell response.

## Discussion

Combination treatment strategies with PD-(L)1 and CTLA-4 ICB antibodies have shown a higher clinical efficacy than either agent alone (5, 66–69) and are currently approved in melanoma and NSCLC. CTLA-4 blockade has an impact on the immune priming phase by inducing the activation and proliferation of effector T cells and by reducing T-reg mediated suppression of T cell responses (69, 70). PD-(L)1 blockade functions during the effector phase to restore immune function in T cells that have high levels of antigen exposure (39, 69, 71). Due to their nonredundant effects on immune cell biology, PD-1 and CTLA-4 blockade have the potential for synergistic effects in the treatment of NSCLC. Despite the therapeutic benefit observed with combination immunotherapies, the majority of patients do not benefit from treatment or initial responders may relapse due to acquired resistance (10, 67, 68, 72).

Here we show that blockade of PD-1 and CTLA-4 resulted in robust tumor suppression with functional augmentation of tumor-infiltrating CD4^+^/CD8^+^ T cells and an antitumor cytokine milieu. But the therapeutic effects of combination ICB were observed during the early phase of tumor growth, with resistance to therapy developing over time. FACS analysis revealed an enrichment of immunosuppressive intratumoral regulatory T cells, neutrophils and Ly6C+ monocytes. In addition, the initial functionally active T cell compartment reverted to an exhausted, non-proliferative state.

To explore the optimal strategy for treating the resistant tumors, we evaluated antibody-based depletion of each of the immunosuppressive immune cell populations. We found that addition of a depleting antibody for CD25+ T cells, GR1 expressing myeloid cells, and Ly6G+ cells significantly inhibited the resistance to anti-PD-1/CTLA-4, but did not produce complete tumor regressions. Strikingly, addition of anti-Ly6C to the anti-PD-1/CTLA-4 combination herein proved to not only prevent resistance from occurring but also produced complete tumor rejection. Further analysis of tumors treated with anti-Ly6C/PD-1/CTLA-4 revealed an accumulation of mature dendritic cells. Dendritic cells are antigen-presenting cells with potent T cell stimulatory capacity that direct the activation and differentiation of T cells by providing co-stimulatory signals (73). Dendritic cells are critical regulators of both innate and adaptive immune responses (38, 74). The combination of anti-PD-1/CTLA-4 was shown to enhance T cell function, however without a sufficiently large number of activated dendritic cells to cross-present and activate the T cells, a sub-optimal anti-tumor effect was observed.

We observed a highly enriched classical monocyte population in the tumors resistant to anti-PD-1/CTLA-4 treatment. Similar observations were made in NSCLC and melanoma datasets with patients treated with ICB in that non-responders/patients with progressive disease have higher level of classical monocytes as compared to responders or patients with response. The monocytes were found to be recruited to the tumor via the CCL2-CCR2 axis in an IFN-γ dependent manner. High infiltration of monocytes with immunosuppressive features have been shown to lead to T cell dysfunction and failure to respond to immunotherapy (75–78). Interestingly, the depletion of the suppressive monocytes with anti-CCL2 did not enhance antitumor response to anti-PD-1/CTLA-4 therapy, but instead blunted the effect.

Addition of anti-Ly6C to the anti-PD-1/CTLA-4 combination prevented resistance from forming and importantly facilitated complete tumor elimination. Our data suggests that anti-Ly6C in the anti-PD-1/CTLA4 combination has an impact predominantly on classical Ly6C+ monocytes. The heterogenous Ly6C monocyte populations have distinct differentiation potential. Classical Ly6C+ monocytes in the immunotherapy resistant tumors have lost their differentiation potential into M1 and dendritic cells, via Flt3/PU.1. The current study delineates the importance of classical monocytes within immunotherapy resistant tumors and potentially how their differentiation can be directed into tumor antigen recognizing dendritic cells. Tumor-infiltrating monocyte-derived dendritic cells have potent antigen-presenting capacity, thereby enhancing the antitumor activity of anti-PD-1/CTLA-4 therapy.

Murine monocytes are heterogenous and consist of a classical/intermediate population Ly6C+CD11b+CD115+ or a nonclassical Ly6C-CD11b+CD115+. Intriguingly, a human homolog for Ly6C has not been reported and the ligands for Ly6C are unknown. Human monocytes are also heterogenous and are made up of classical CD14++CD16-, intermediate CD14+CD16+, or nonclassical CD14+CD16++ monocytes (79, 80). Single cell transcriptomics analysis of tumor-infiltrating myeloid cells in humans and mouse lung cancers revealed that monocytes are conserved across the species with evidence of a one-to-one equivalence (15). Nonetheless the identification of a Ly6C human homolog or orthologue, or a better understanding of the monocyte population(s) defined by Ly6C expression for which there are human counterparts, is warranted to better define how these cell types can be directed as possible treatment options to mitigate ICB therapy resistance.

In addition, a better understanding of the blocking antibody anti-Ly6C Monts1 and how it binds to Ly6C on the surface of classical monocytes and changes differentiation potential will be necessary. Blocking antibodies are known to bind to ligand or receptor on the surface of cells and block the target signaling pathway. For example, PD-1 pathway blockade restores activity of antitumor T cells that have become quiescent by preventing the binding of PD-1 to PD-L1. Blockade with anti-Ly6C could be potentially preventing binding of a ligand to Ly6C on the cell surface of monocytes. It is also possible that the blocking Ly6C antibody may also block Ly6C on other immune cell types including neutrophils, macrophages, and MDSC. Better understanding of the Ly6C interactome will help uncover interactions that are critical for monocyte development and are currently under investigation.

Myeloid cell subsets can circumvent T cell directed ICB therapies by reducing antigen presentation and attenuation of T cell activation, thereby leading to impaired antitumor immune response (12–14, 81). ICB aimed at restoring T cell function only partially addresses the immune dysfunction in the tumor microenvironment. It is also essential to modulate the myeloid populations in order to produce a durable anti-tumor immune response. Our study demonstrates that monocytes, a myeloid cell subset, are recruited into inflamed tumor tissues and proliferate after anti-PD-1/CTLA-4 treatment, where they can mediate suppressor roles or rapidly differentiate to dendritic cells upon Ly6C blockade. These TMDCs can drive T-cell activation and proliferation, thereby enhancing the antitumor immunity. This study not only delineates a previously unrecognized mechanism of monocyte-to-dendritic cell differentiation in the immunotherapy resistant tumor microenvironment but also provides a potential strategy to optimize immunotherapy for cancer.

## Data availability statement

The authors confirm that the data supporting the findings in the results are available within the article and the supplementary material. Raw data from this study are available from the corresponding author upon request. 

## Ethics statement

The animal studies were reviewed and approved by The University of Texas MD Anderson Cancer Center Animal Care and Use Committee.

## Author contributions

BR, LC, and DG conceived and designed the study. LC, YL, BR, DP, SM, and XY performed and interpreted the experiments. LD developed and performed bioinformatics and statistical analysis. JF, LG, JK, JO, AP, FA, LS, and XZ helped with experiments. LC, JW, and DG supervised the participants. BR, LC, and DG wrote/edited the manuscript. All authors contributed to the article and approved the submitted version.

## Supplementary material

Supplementary Figure 1
**(A)** The short-term treatment of anti-PD-1_CTLA-4 changes the profile of cytokines/chemokines in tumors. Tumors from **Figure 1A**, anti-PD-1 (200 µg per mouse), anti-CTLA-4 (200 µg per mouse), combination (200 µg of anti-PD-1 plus 200 µg of anti-CTLA-4), or their IgG control mixture was intraperitoneally injected into Sv129 mice once a week for 3 weeks beginning on day 7 after 344SQ lung cancer cells were subcutaneously implanted (0.1 x 10^6^ cells per mouse). Tumors were measured once a week for 4 weeks, were harvested at the end point. 1/3 of each individual tumor was prepared for tumor lysate. The concentration of IFN-α, IFN-γ, TNF-α, TGF-β, IL4, IL27, M-CSF, GM-CSF, and CCL2 in tumors were measured by ELISA assay. The data were summarized and shown. The ANOVA test was used to analyze the data. ns, no significant difference; *p < 0.05; **p < 0.01; ***p < 0.001; ****p < 0.0001.

Supplementary Figure 2 Kaplan-Meier survival curves from 344SQ tumors from **Figure 2E** **(A)** survival curve from 393P tumors from **Figure 2F** **(B)** survival curve from 344P tumors from **Figure 2G** **(C)** survival curve from 412P tumors from **Figure 2H** **(D).** *p* values were calculated with *t*-test. n.s., no significant difference; **p* < 0.05; ***p* < 0.01; ****p* < 0.001; *****p* < 0.0001.

Supplementary Figure 3 Dual treatment of anti-Ly6C_PD-1 or anti-Ly6C_CTLA-4. **(A)** The dual blockade of Ly6C_PD-1 or Ly6C_CTLA-4 does not result in a curative therapeutic effect. 344SQ tumor bearing 129/Sv mice were weekly treated with anti-Ly6C alone (200 µg of anti-Ly6C per mouse), anti-Ly6C_PD-1 (200 µg of anti-Ly6C plus 200 µg of anti-PD-1 per mouse), anti- Ly6C_CTLA-4 (200 µg of anti-Ly6C plus 200 µg of anti-CTLA-4 per mouse), or their IgG control mixture (IgG control) beginning on day 7 after a subcutaneous cancer cell injection (1 x 10^6^ cells per mouse; n = 5) for 4 weeks. The tumor growth was weekly monitored. The tumor growth curves are shown in the left panel. At week 6, mice were euthanized to measure the primary tumor weight (middle) and metastatic lung nodules (right). At the end point, tumors were analyzed by FACS for immune profiling **(B).** The summarized data with ANOVA test is shown in. ns, no significant difference; *p < 0.05; **p < 0.01.

Supplementary Figure 4 Monts-1 binds to Ly6C but does not deplete the Ly6C+ population **(A)** Ly6C or empty control vector expression in HEK293T cells were evaluated for EGFP expression (top) or Monts-1 Ly6C AF647 and HK1.4 Ly6C APC-Cy7 (bottom). Ly6C or empty control vector expression in HEK293T cells were evaluated for EGFP expression (top) or Monts-1 Ly6C AF647 and AL-21 Ly6C PE (bottom). **(B)** 129/Sv mice were treated with 200 µg of anti-Ly6C (Monts 1) per mouse or the IgG control via IP injection once a week for 4 weeks. The Ly6C^+^CD11b^+^ population in spleens was analyzed by FACS. The staining antibody for Ly6C is HK1.4. The representative plots are shown.

Supplementary Figure 5 344SQ tumors from **Figure 3A** were FACS analyzed for M1 macrophage or M2 macrophage markers. **(B)** H&E stained lung sections at week 8 from anti-PD-1/CTLA-4 or anti-PD-1/CTLA-4/Ly6C treated mice from **Figure 3E**. Scale bar= 5 mm. **(C)** IHC of representative CD8 staining in lung tumor from mice treated in **Figure 3E**, quantitation shown in **Figure 3I**.

Supplementary Figure 6
**(A)** C57BL/6 mice were treated weekly with combined anti-PD-1 and anti-CTLA-4 (Combination: 200 mg of anti-PD-1 plus 200 mg of anti-CTLA-4 per mouse) or their IgG control mixture (IgG control) beginning on day 7 after a subcutaneous melanoma B16 cell injection (0.1 x 10^6^ cells per mouse; n = 5) for 8 weeks. FACS analysis of percentage of CD14+CD45+ monocytes quantitated from B16 at week 9. **(B)** B16-OVA (B16-expressing OVA) melanoma cells (2 × 10^5^ per mouse) were subcutaneously injected into 6-8 week old C57BL/6 mice. On day 7, the mice were treated with IgG control or anti-PD-1_CTLA-4 (200 mg of anti-PD-1 plus 200 mg of anti-CTLA-4 per mouse) once a week for three times. In the treatment of dendritic cell transfer plus anti-PD-1_CTLA-4 (DC + anti-PD-1_CTLA-4) group, the TMDCs were pulsed with 10 µg/ml OVA overnight and injected via tail vein (1 × 10^5^ per mouse) on day 7 and 14. BSA-treated DCs were used as the control. The tumor growth curves and tumor weights at the end point are shown. The mature dendritic cells in tumors were analyzed by FACS and are shown on right. **(C)** The tumor lysates were subjected to ELISA for measuring cytokine concentrations in tumors. **(D)** 393P tumor bearing 129/Sv mice were treated weekly with triple blockade of PD-1, CTLA-4, and Ly6C (Tritherapy: 200 µg of anti-PD-1 plus 200 µg of anti-CTLA-4 plus 200 µg of anti-Ly6C per mouse) or their IgG control mixture (IgG control) beginning on day 7 after a subcutaneous cancer cell injection (0.1 x 10^6^ cells per mouse; n = 5) for 4 weeks. For blocking B7 signal, antibodies (anti-B7: 300 μg of anti-CD80 and 300 μg of anti-CD86 per mouse) were intraperitoneally administered 1 day before the first dose of therapy, and then once a week to maintain the blockade.

Supplementary Figure 7
**(A)** Classical monocytes and non-classical monocytes from NSCLC nivolumab treated patients (GSE126044). **(B)** 344SQ tumor bearing 129/Sv mice were weekly treated with the anti-CCL2 alone (100 mg of anti-CCL2 per mouse), the combined anti-PD-1 and anti-CTLA-4 (anti-PD-1_CTLA-4: 200 mg of anti-PD-1 plus 200 mg of anti-CTLA-4 per mouse), the combined anti-PD-1, anti-CTLA-4, and anti-CCL2 (anti-PD-1_CTLA-4_CCL2: 200 mg of anti-PD-1 plus 200 mg of anti-CTLA-4 plus 100 mg of anti-CCL2 per mouse), or their IgG control mixture beginning on day 7 after cancer cell subcutaneous injection (0.2 x 10^6^ cells per mouse; n = 5) for 5 weeks. Tumors were measured once a week. The tumor growth curves, and primary tumor masses are shown. At the end point, tumors were harvested to prepare single cell suspensions. The single cell suspensions were subjected to FACS analysis. The tumor-infiltrating monocyte populations **(C)** and mature dendritic cells (mDCs) **(D)** and the CD4 and CD8 T cells **(E)** from different groups are shown. The results are shown with *t*-test. ns, no significant difference; *p < 0.05; **p < 0.01; ***p < 0.001; ****p < 0.0001. In (F, J, and K), the results are shown with ANOVA test. ns, no significant difference; *p < 0.05; **p < 0.01; ****p < 0.0001.

Supplementary Figure 8
**(A)** Heat map of association between the mRNA levels of human monocyte gene signature and IFNγ pathway in the BATTLE-2 and PROSPECT datasets. Spearman correlation with adjusted p value < 0.05 was used as the criteria to select the most significant IFN-γ-related markers for generating the heat map. **(B)** Spearman’s rank correlation (Rho) was used to assess the association between CCL2/CCR2 and human monocyte marker expression in lung cancer patients’ samples from BATTLE-2 and PROSPECT datasets.

Supplementary Figure 9
**(A)** Gating scheme used to characterize R1, R2, and R3 Ly6C+ monocytes shown in **Figure 5E**. **(B)** (Left) Percentage of Nur77 on monocytes from 344SQ tumors treated with IgG or anti-PD-1/CTLA-4 combo until week 7. (Right) qPCR of Nr4a from Ly6C+ or Ly6C- FACS sorted monocytes from IgG CTL or combo (anti-PD-1/CTLA-4) treated tumors.

Supplementary Figure 10 FACS analysis gating scheme.

Supplementary Table 1 Table of antibodies used for flow cytometry and primers used for qPCR’s.
