# Supplementary material for: Targeting immunosuppressive Ly6C+ classical monocytes reverses anti-PD-1/CTLA-4 immunotherapy resistance
Source: Front Immunol. 2023 Jun 28;14:1161869. doi: 10.3389/fimmu.2023.1161869 (PMC10336223; doi:10.3389/fimmu.2023.1161869)
Supplement: Supplementary Figure 1 — (A) The short-term treatment of anti-PD-1_CTLA-4 changes the profile of cytokines/chemokines in tumors. Tumors from Figure 1A , anti-PD-1 (200 µg per mouse), anti-CTLA-4 (200 µg per mouse), combination (200 µg of anti-PD-1 plus 200 µg of anti-CTLA-4), or their IgG control mixture was intraperitoneally injected into Sv129 mice once a week for 3 weeks beginning on day 7 after 344SQ lung cancer cells were subcutaneously implanted (0.1 x 10^6^ cells per mouse). Tumors were measured once a week for 4 weeks, were harvested at the end point. 1/3 of each individual tumor was prepared for tumor lysate. The concentration of IFN-α, IFN-γ, TNF-α, TGF-β, IL4, IL27, M-CSF, GM-CSF, and CCL2 in tumors were measured by ELISA assay. The data were summarized and shown. The ANOVA test was used to analyze the data. ns, no significant difference; *p < 0.05; **p < 0.01; ***p < 0.001; ****p < 0.0001. [file Presentation_1.pptx]

## Slide 1
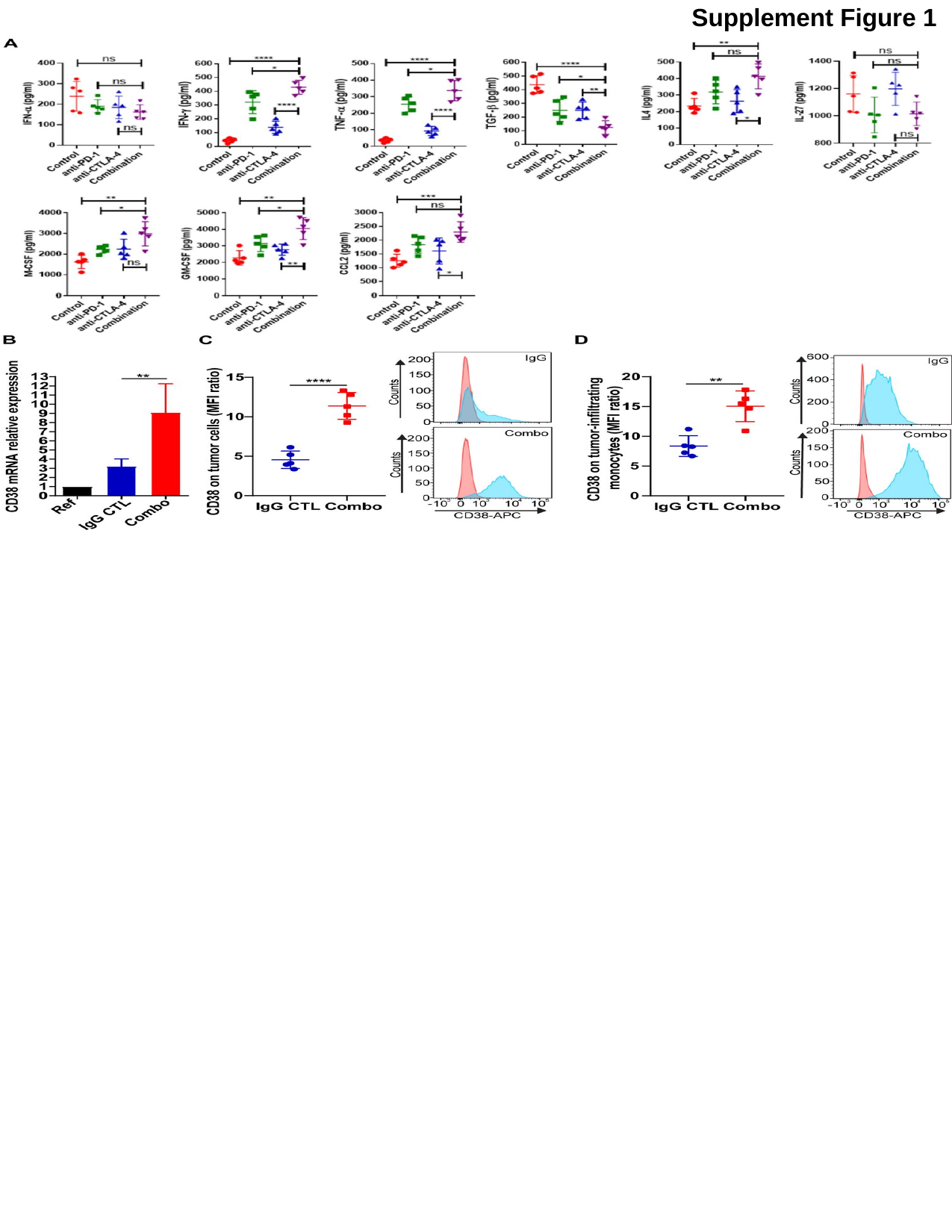

Supplement Figure 1

## Slide 2
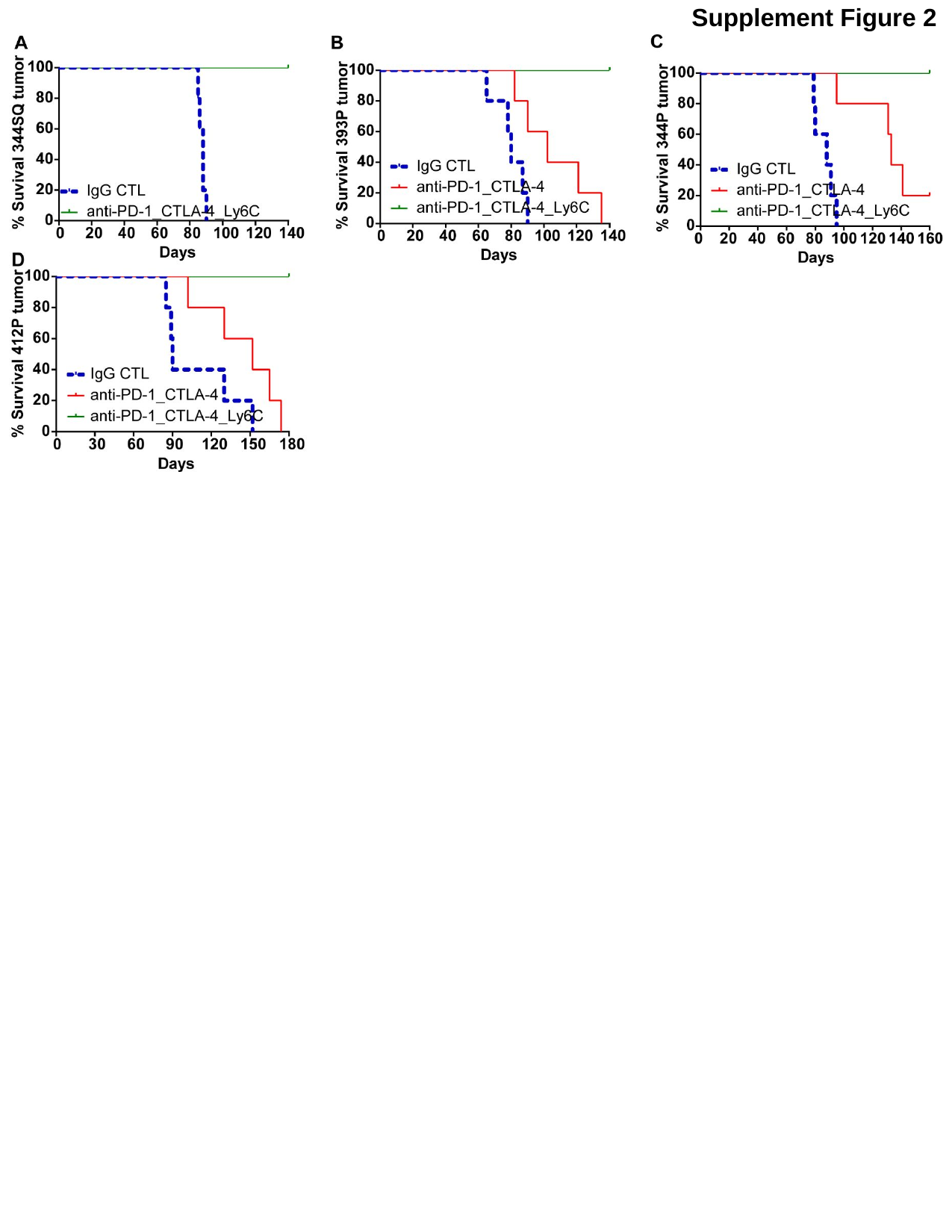

Supplement Figure 2

## Slide 3
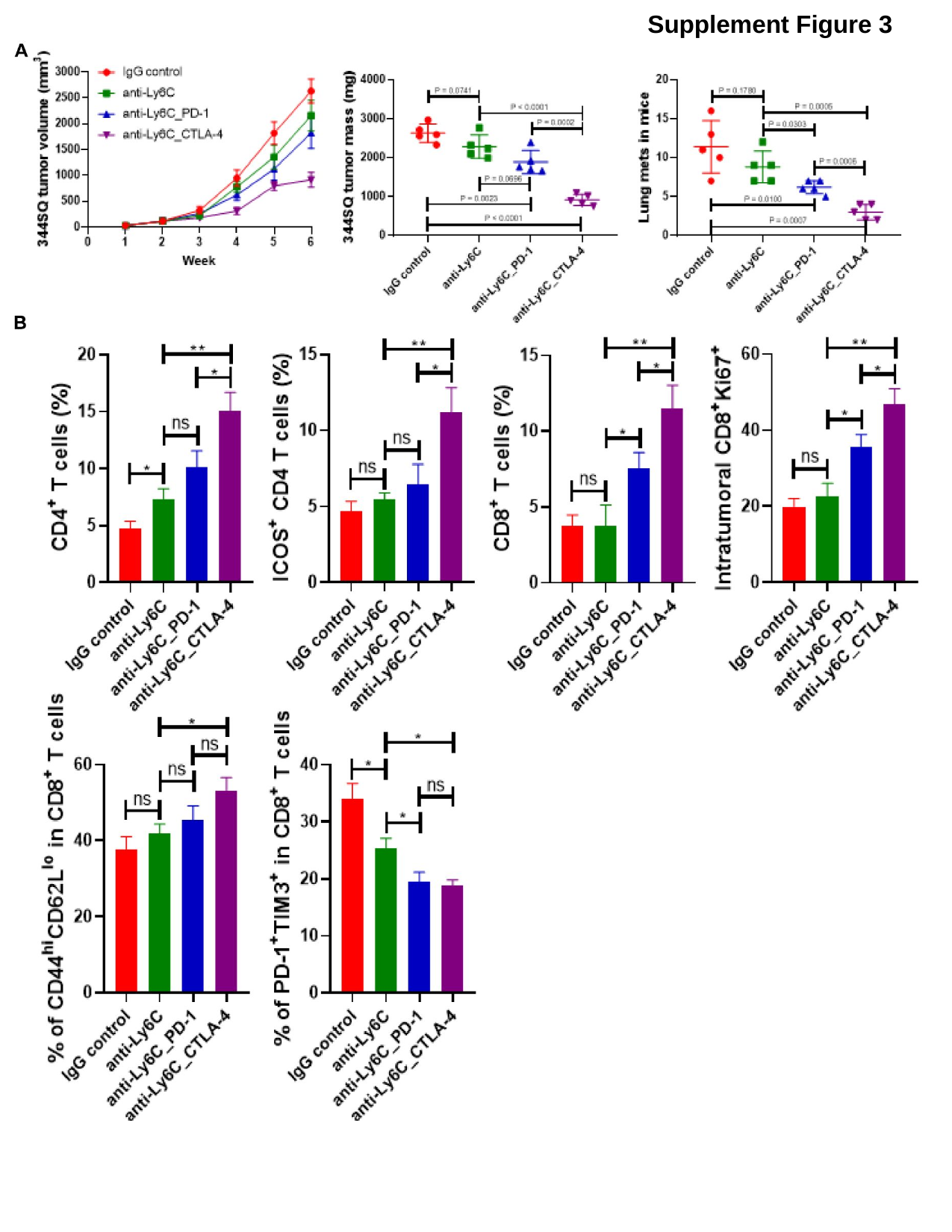

Supplement Figure 3

## Slide 4
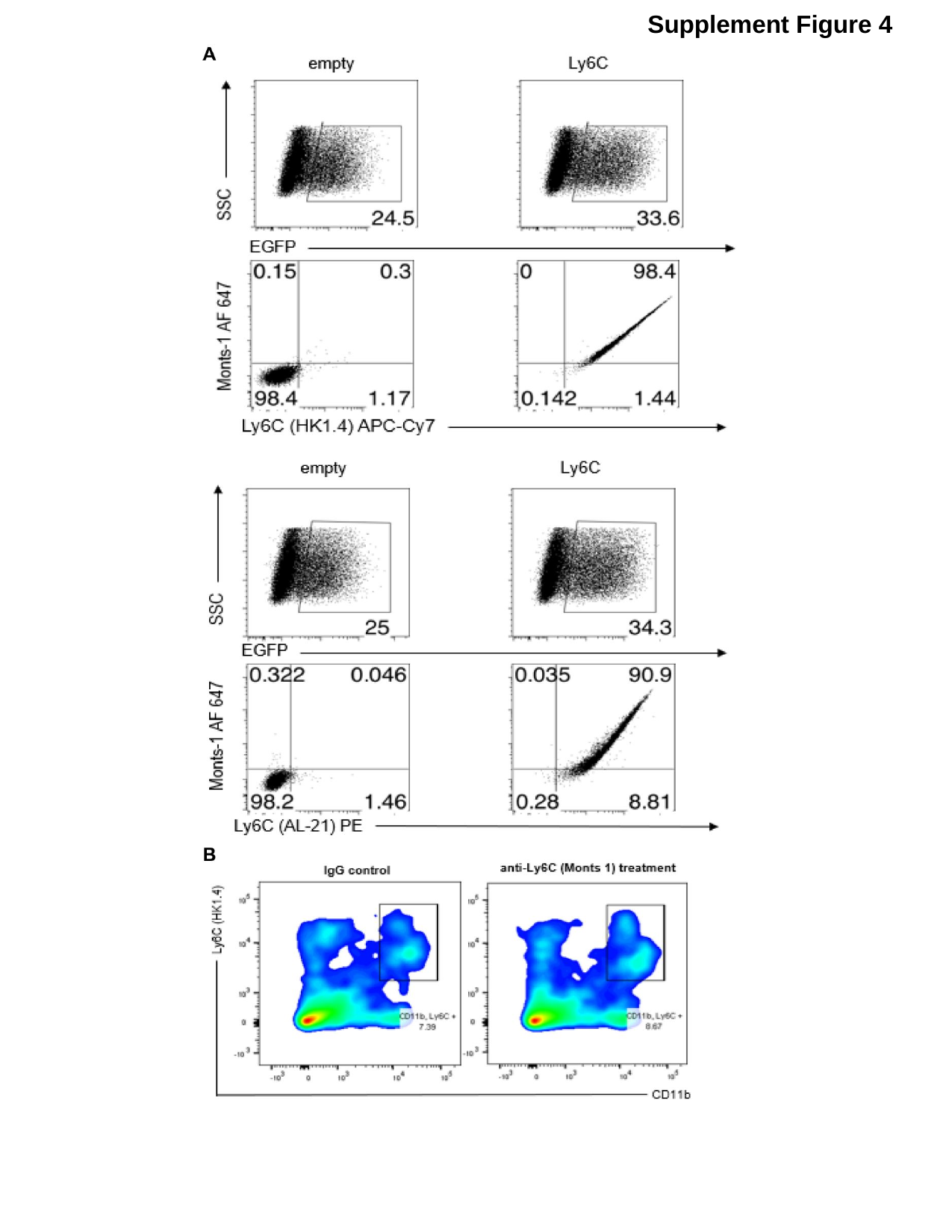

Supplement Figure 4

## Slide 5
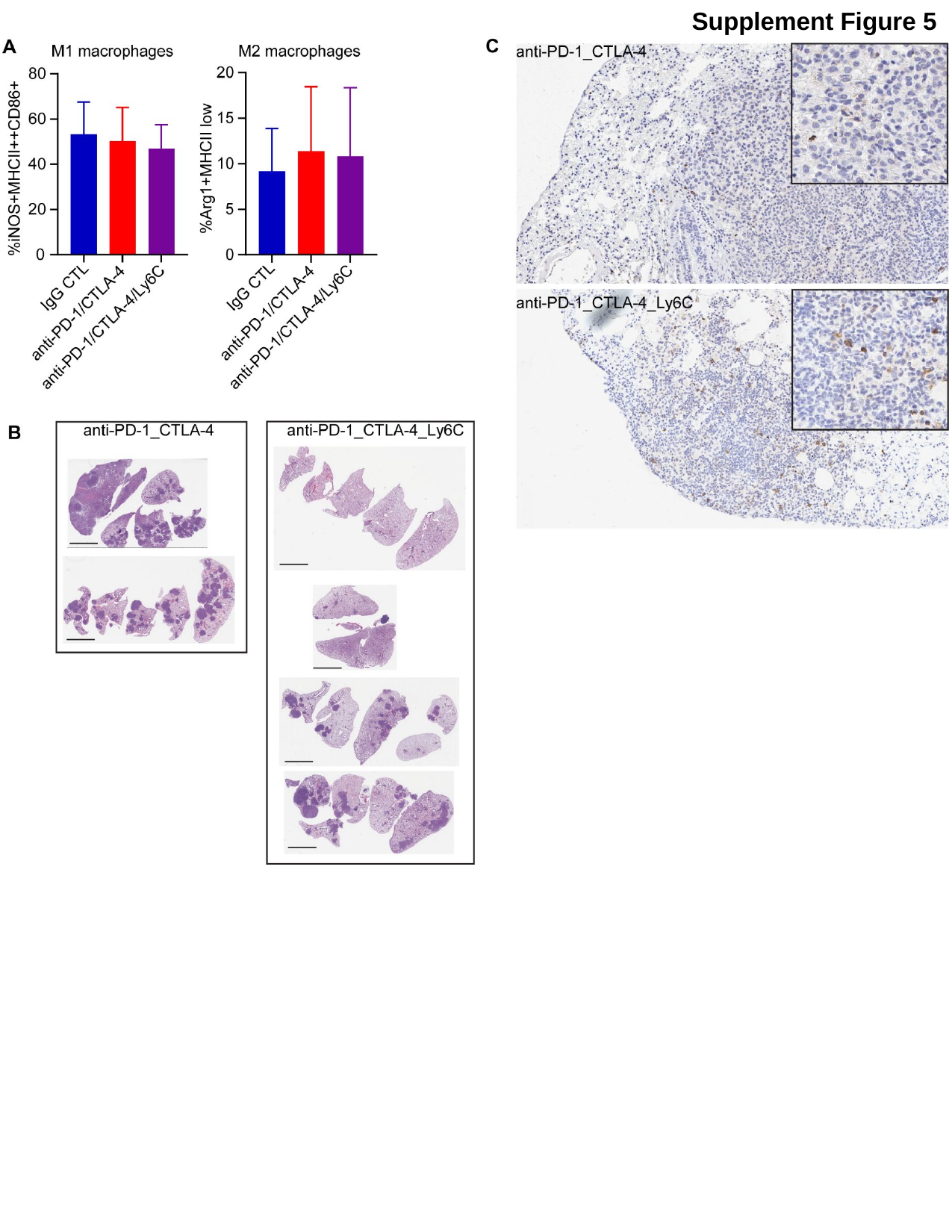

Supplement Figure 5

## Slide 6
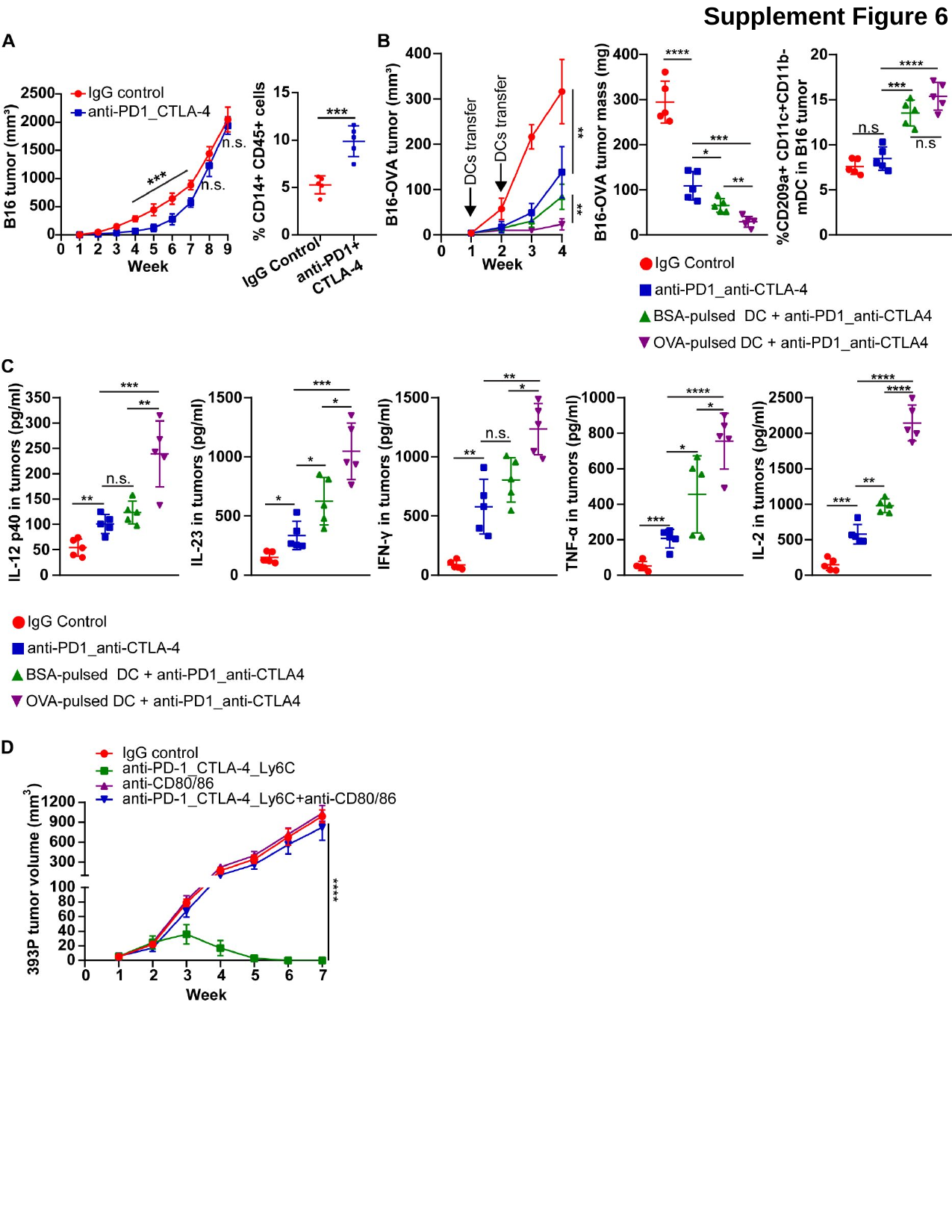

Supplement Figure 6

## Slide 7
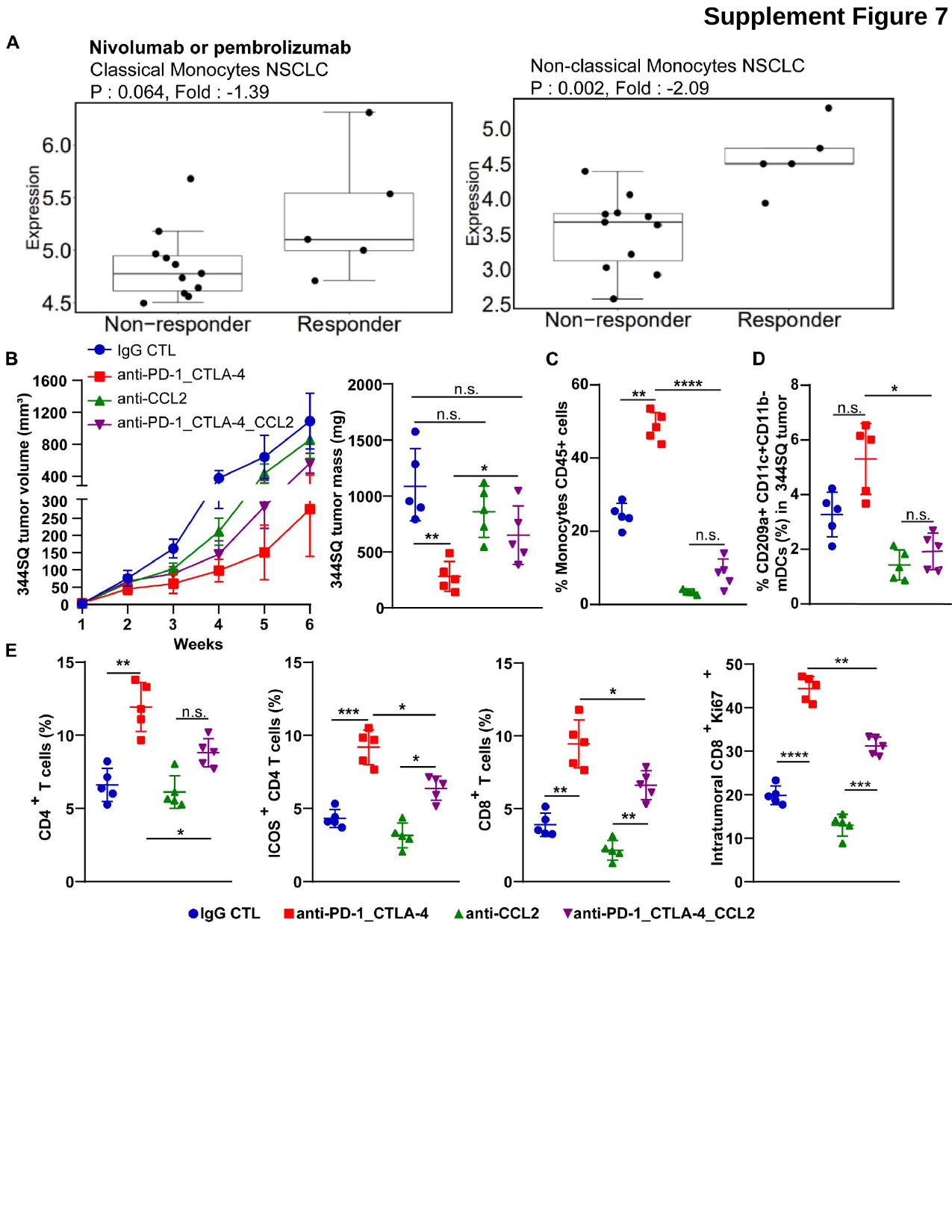

Supplement Figure 7

## Slide 8
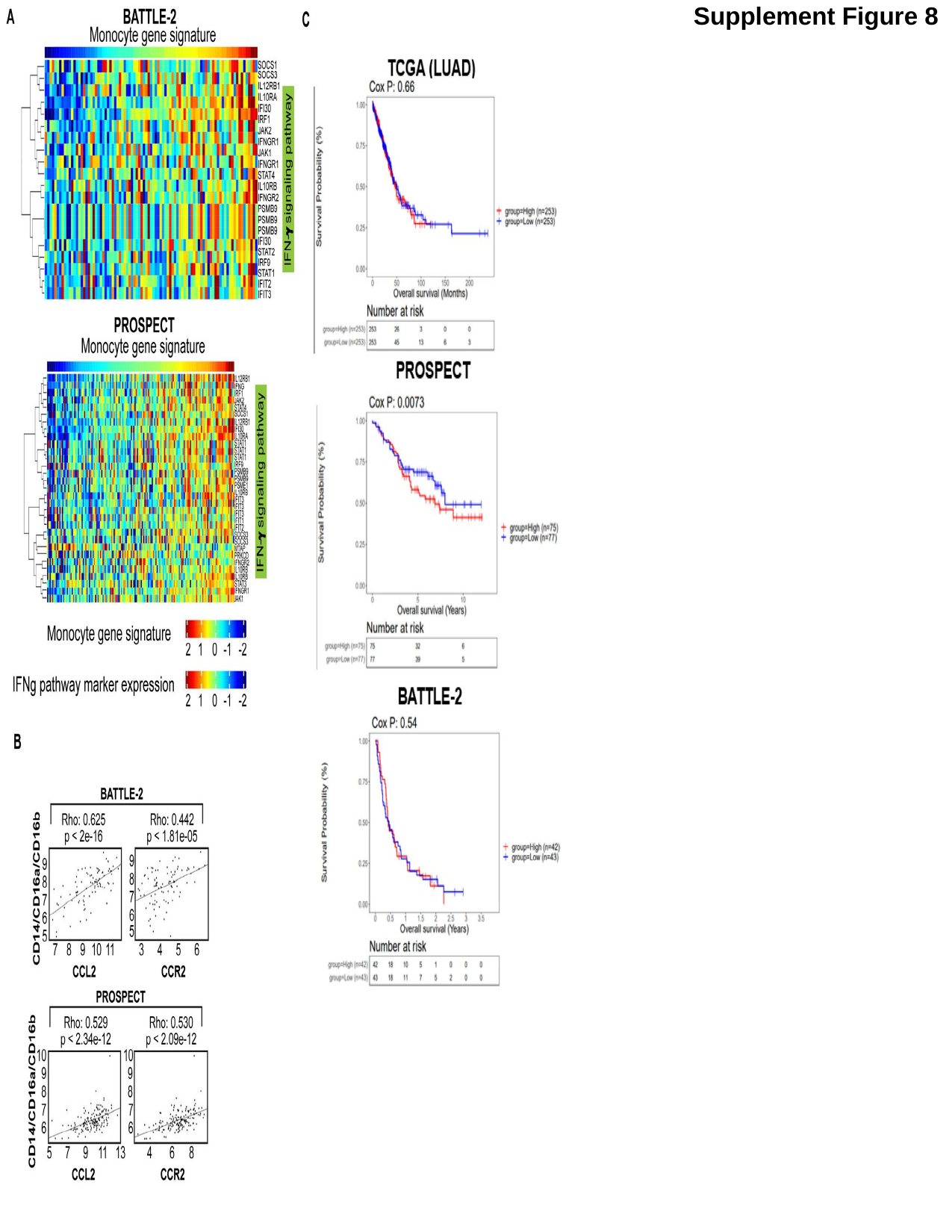

Supplement Figure 8

## Slide 9
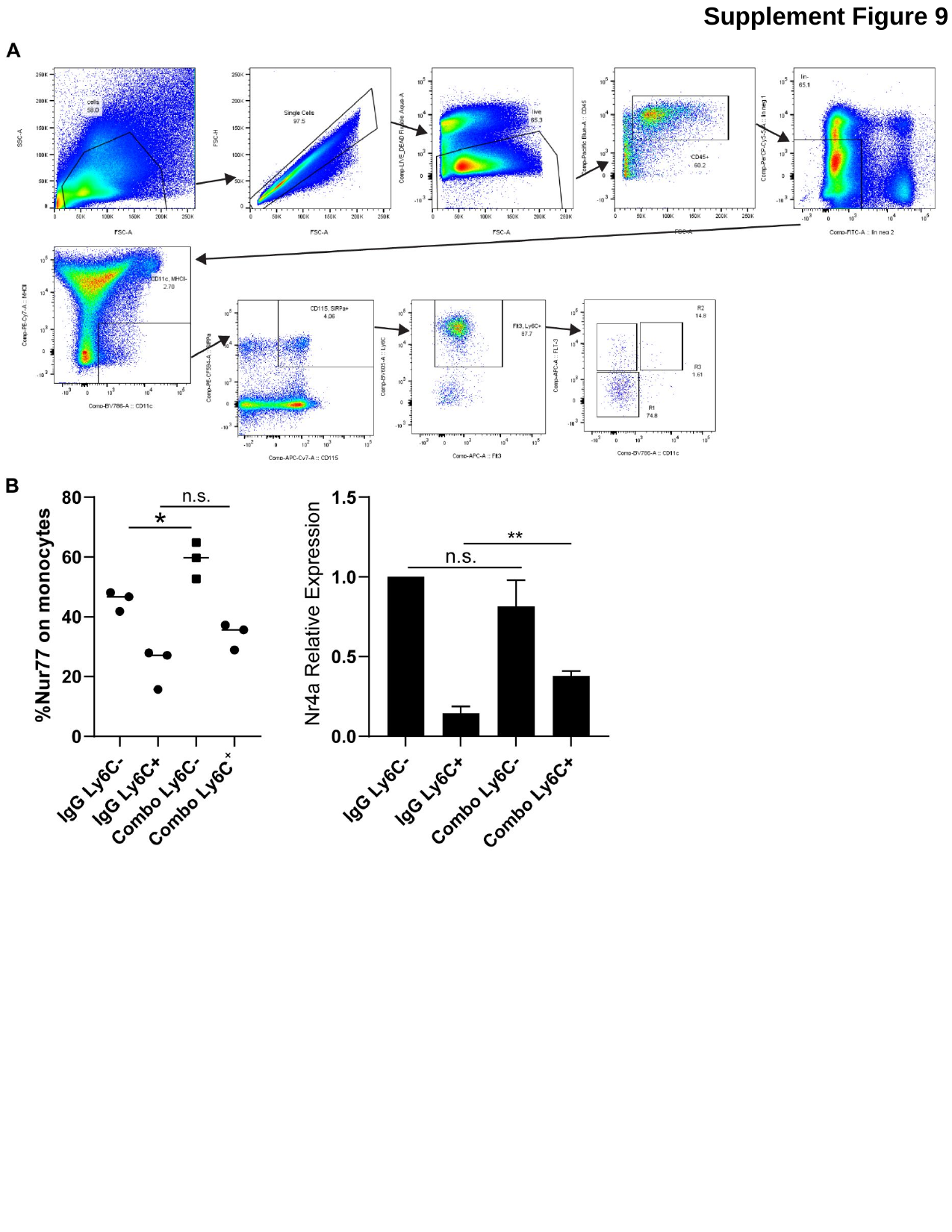

Supplement Figure 9

## Slide 10
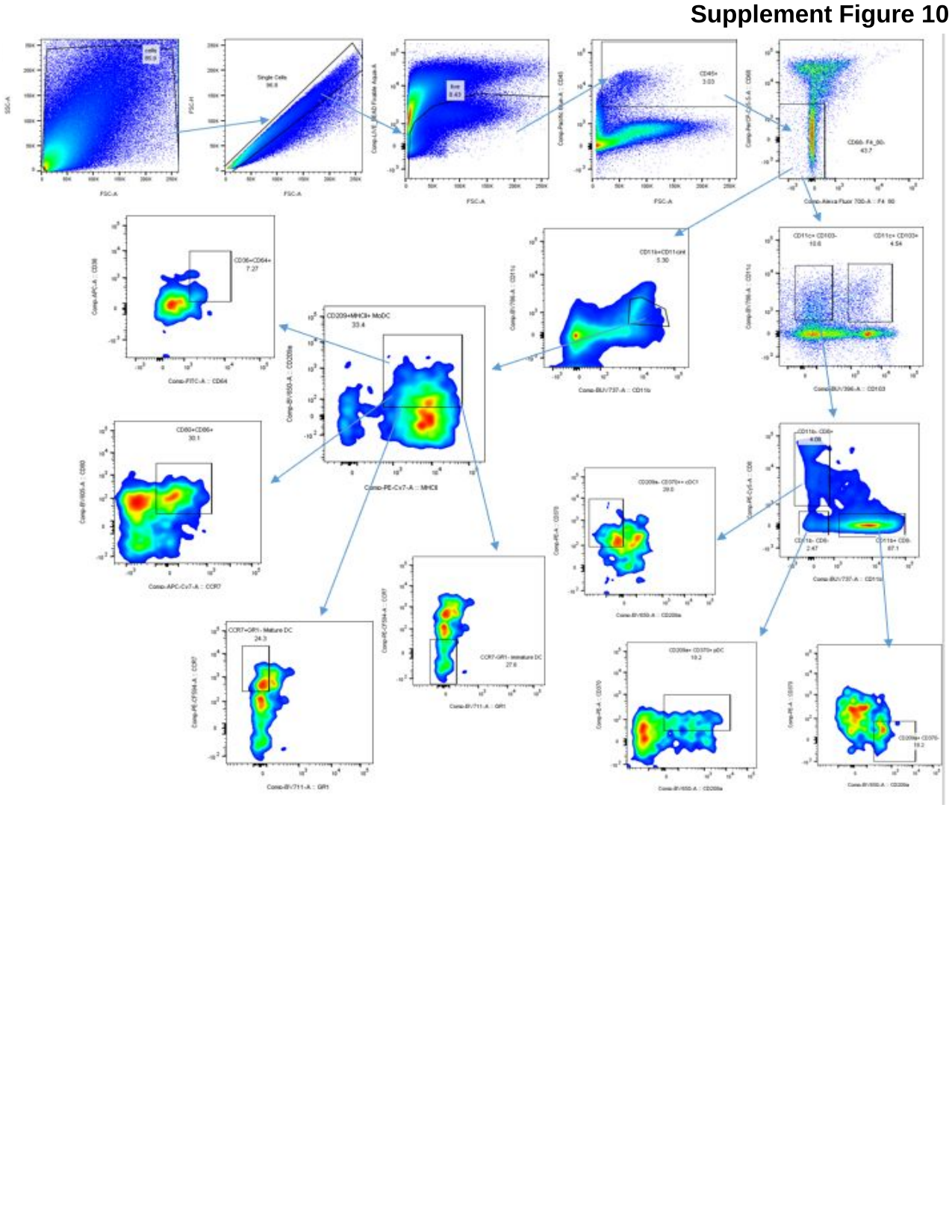

Supplement Figure 10

## Slide 11
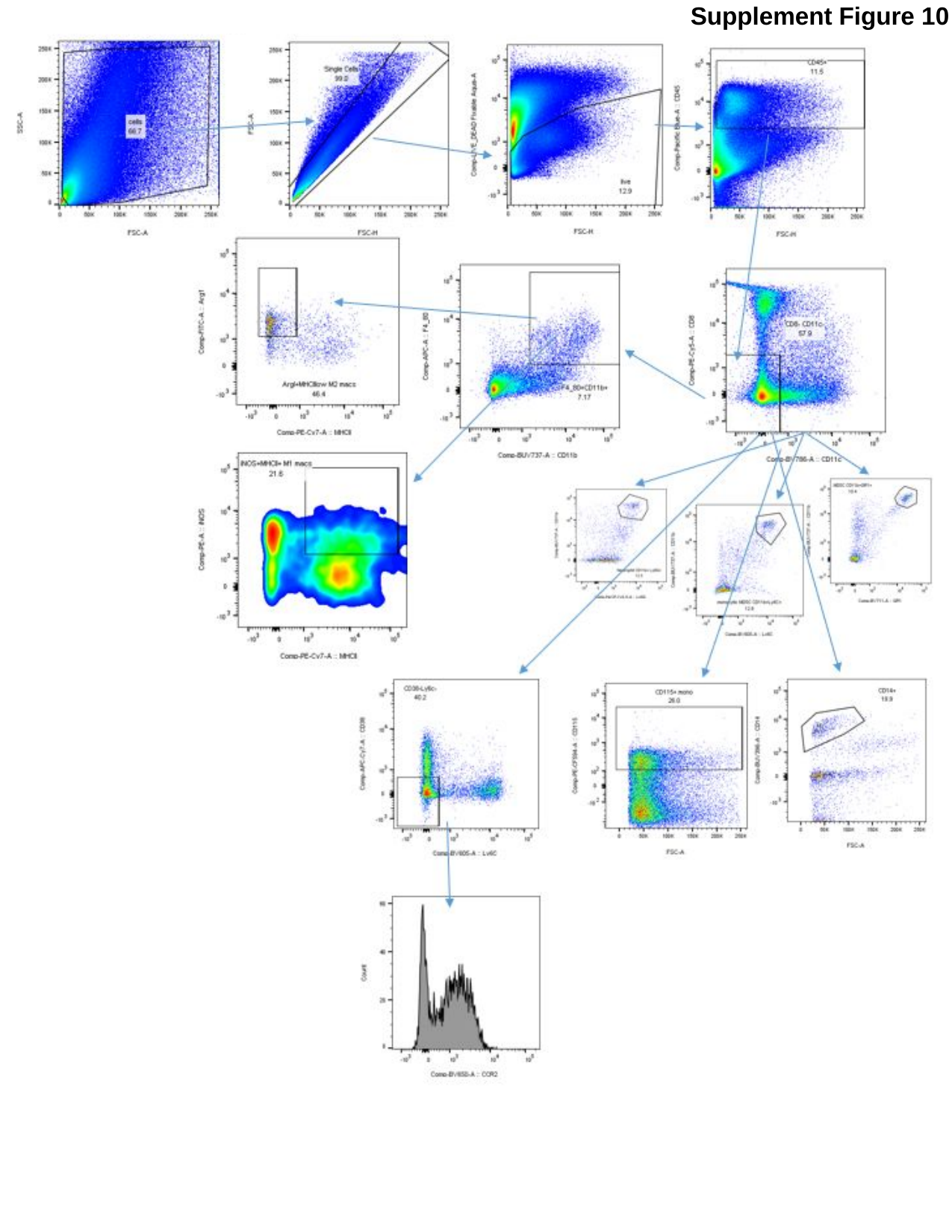

Supplement Figure 10
